# OFC-induced network modularity improves positive symptoms and attentional alertness in schizophrenia: a combined rTMS-fMRI study

**DOI:** 10.1038/s41467-026-72917-4

**Published:** 2026-05-30

**Authors:** Ningning Zeng, Min Wang, Hui Zheng, Xiong Jiao, Ziliang Wang, Kexu Zhang, Katharina S. Goerlich, André Aleman, Jijun Wang, Qiang Hu

**Affiliations:** 1 Neuroregulation Center, Wuhu Hospital of Anding Hospital (The Fourth People’s Hospital of Wuhu), Wuhu, China; 2 University Medical Center Groningen, University of Groningen, Groningen, Netherlands; 3 Department of Psychology, Ningbo University, Ningbo, China; 4 Department of Psychology, School of humanities and social sciences, University of Science and Technology of China, Hefei, China; 5 Shanghai Key Laboratory of Psychotic Disorders, Shanghai Mental Health Center, Shanghai Jiao Tong University School of Medicine, Shanghai, China; 6 Shanghai Med-X Engineering Research Center, School of Biomedical Engineering, Shanghai Jiao Tong University, Shanghai, China; 7 Department of Psychiatry, Zhenjiang Mental Health Center, Zhenjiang, China; 8 Department of psychiatry, Shandong Daizhuang Hospital, Jining, China; 9 Faculty of Psychology and Neuroscience, Maastricht University, Maastricht, Netherlands; 10 Mental and Psychological Rehabilitation Research Center, Center of Yuanshen Rehabilitation Institute, Shanghai Jiao Tong University School of Medicine, Shanghai, China; 11 Nantong Fourth People’s Hospital & Nantong Brain Hospital, Nantong, China; 12 Department of Psychiatry, School of Medicine, Jiangsu University, Zhenjiang, China

**Keywords:** Predictive markers, Randomized controlled trials, Schizophrenia

## Abstract

Repetitive Transcranial Magnetic Stimulation (rTMS) targeting the orbitofrontal cortex (OFC) has emerged as a promisingerapeutic option for drug-naïve people with schizophrenia (SCZ). However, the putative underlying mechanisms of OFC-induced physiological effects remain unknown. In this completed randomized, double-blind, placebo-controlled trial (ChiCTR2000041106), we delivered 4 weeks of low-frequency rTMS to the right OFC in SCZ, with participants receiving either active or sham stimulation, and followed a network neuroscience framework to explore the alteration of dynamic modularity induced by the OFC. The trial met its pre-specificized primary endpoint following active treatment. Neuroimaging analysis reported here were secondary outcomes. We found that the modularization between OFC and the default mode network (DMN) across time windows supported improvements in symptoms and cognitive function. This dynamics pattern was spatially constrained, with stronger rTMS modulation observed in DMN regions centered on the ventromedial prefrontal cortex (vmPFC). The spatial topography of this pattern was correlated with the expression of schizophrenia-related genes and markers of excitatory neurotransmission, supporting its biological relevance. Crucially, such cascade of physiological effects was specifically linked to improvements in cognitive attention/vigilance and were modulated by their positive symptoms. Exploratory analyses showed that OFC-induced modularity weakened the DMN’s causality over the downstream attention network. These findings reveal the important role for the dynamic modularity of the OFC as an “intermediate phenotype” mediating the pathway from genetic variation to behavioral manifestations, highlighting the potential of low-frequency stimulation of the OFC as a therapeutic strategy for specific subgroups of SCZ, especially those with positive symptoms and attention deficits.

## Abstract (web summary)

In this study, the authors identify a network mechanism where rTMS targeting the orbitofrontal cortex improves attentional vigilance and positive symptoms in patients with first episode schizophrenia.

## Introduction

Schizophrenia (SCZ) is one of the most complex and chronic psychiatric disorders^1,2^. Despite appropriate pharmacotherapy treatment of schizophrenia, it remains unsatisfactory in reducing negative symptoms and the propensity to relapse^3,4^. Uncovering the etiology and pathogenesis and developing more effective and acceptable treatments remains one of the most formidable challenges facing psychiatry and neuroscience. With the emergence of the perspective of neurodevelopmental disorders, aberrant brain connectivity has been postulated as an important pathophysiological mechanism underlying schizophrenia^5–9^. Abnormalities of structural and functional network architecture may ultimately manifest as a diverse range of disturbances of positive and negative symptoms, as well as neurocognitive deficits^5,6,9^.

Transcranial magnetic stimulation (TMS), a noninvasive brain stimulation technique, has played a promising role in modulating brain activity and clinical symptoms^10–14^. It serves as a reliable framework for explaining, comprehending, modifying, and potentially preventing brain-related outcomes of interest^11^. Clinically, rTMS targeting the prefrontal cortex has been approved by the US Food and Drug Administration (FDA) as a treatment for certain psychiatric conditions, most notably major depressive disorder. Accumulating evidence suggests the efficacy of repetitive TMS (rTMS) in treating schizophrenia based on different stimulation targets like the dorsolateral prefrontal and temporoparietal cortex^10,12^. However, its clinical outcomes remain variable, and not everyone experiences symptom relief^13,14^. Proposed explanations range from variations in stimulation parameters and targeted brain areas to individual differences in symptom severity and neural connectivity^13,15^. This heterogeneity poses a challenge for both clinical practice and research. Intuitively, different stimulation targets should elicit specific neural pathway effects, thereby tracking improvements in specific symptoms and cognitive factors. To date, there is only limited evidence demonstrating either the neural specificity of rTMS physiological effects in the human cortex or the symptom specificity of neural modulation in SCZ^16,17^.

The orbitofrontal cortex (OFC), a critical hub for cognitive and affective processes due to its unique anatomical and functional connectivity, has been proposed as a novel clinical target for rTMS treatment by inhibiting the right part to modulate non-rewarding signaling or expected reward identity^18–21^. It receives direct inputs from subcortical areas and projects to several cortical and subcortical areas and has been suggested to be of key importance in psychiatric disorders^22–24^. A series of studies has demonstrated encouraging results for OFC-rTMS. Inhibitory OFC-rTMS has shown promise in the treatment of obsessive-compulsive disorder (OCD) and substance use disorders (SUD), with studies reporting associated reductions in activation of disorder-relevant brain regions^20,21^. Notably, Fettes et al. first reported a case of major depression resistant to conventional rTMS over the dorsolateral prefrontal cortex (DLPFC) and the dorsomedial prefrontal cortex (DMPFC) but showed marked improvement in mood, anxiety and anhedonia following 1 Hz right OFC-rTMS, accompanied by a reduction in functional connectivity between the OFC and the nucleus accumbens^18^. Subsequent work has further substantiated the safety, tolerability, and clinical outcomes of this protocol in a review^19^. This raises the question of whether OFC-induced physiological effects can be used to reliably improve symptoms and cognitive function in SCZ. Our recent TMS/EEG study demonstrated a reduced EEG response to right OFC stimulation in SCZ^25^. However, the mechanistic effects on network modularity remain to be investigated. Crucially, the physiological effects of rTMS have also been shown to conform to brain dynamics, propagating to “downstream” areas through neural circuits^26,27^. Thus, taking an integrated framework that links the dynamic interaction between OFC and downstream networks may provide a promising indicator for tracking specific modulation and improving the sensitivity of rTMS.

Combining rTMS with fMRI provides a robust experimental manipulation approach, enhancing our understanding of how stimulation paradigms influence distributed activity across distant brain regions. This study aimed to shed light on the specific relationships among OFC-induced physiological effects, in relation to symptom and cognition changes. To accomplish this, we used neuroimaging data acquired from a 4-week randomized, double-blind, and controlled trial (RCT) with rTMS targeting the right OFC on SCZ^25,28^. We recently reported the changes in clinical symptoms and cognitive function in the active rTMS group before and after the intervention: the active group improved to a greater extent than the sham-control group on both psychotic symptoms and cognitive functioning. We next sought to identify the pathways that allow OFC stimulation to affect downstream functional network integration. We used a network neuroscience approach to characterize the dynamic communications between right OFC and functional networks and hypothesized that specific communications would be modulated by distance to the rTMS target. Furthermore, to determine why OFC’s communication varies across the brain, we investigated how OFC’s communication is shaped by underlying genetic factors in SCZ. By spanning multiple levels of analysis across genes, brain connectome, cognition, and symptoms, we ultimately expect to interrogate causal links between OFC-induced physiological effects and specific disease factors.

## Results

### Participants

We recruited a total of 98 subjects from the Psychiatric Department of the First Psychiatric Hospital of Harbin. All subjects were diagnosed with schizophrenia according to the structured clinical interview for DSM-IV. Subjects were randomly assigned to the active rTMS (*n* = 53) or sham rTMS (*n* = 45) group with matched age and sex. Fourteen subjects (8 subjects in the active group and 6 subjects in the sham group) could not complete the clinical assessment, cognitive assessment, or MRI scanning; thus, 84 subjects (45 subjects in the rTMS group (20 females and 25 males) and 39 subjects (21 females and 18 males) in the sham group) were included in total.

**Fig. 1:**
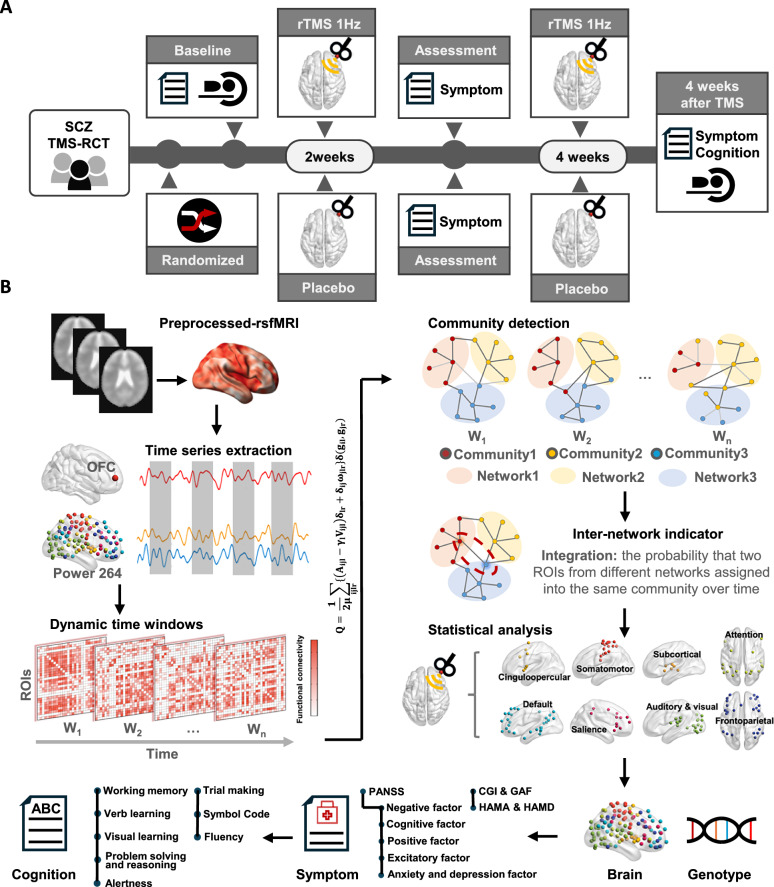
Study design and data analysis pipeline. **A** Flowchart of the randomized controlled trial (RCT) design. Individuals with schizophrenia (SCZ) were randomized to receive either 4 weeks of active 1 Hz repetitive transcranial magnetic stimulation (rTMS) targeting the right orbitofrontal cortex (OFC) or a sham stimulation. Clinical symptoms were assessed at baseline, 2 weeks, and 4 weeks. Cognitive function and resting-state fMRI were assessed at baseline and 4 weeks. **B** Schematic of the neuroimaging data processing and analysis pipeline. Resting-state fMRI time series were extracted from Power 264 atlas and OFC target. Dynamic functional connectivity matrices were constructed using a sliding time-window approach. A multilayer community detection algorithm was then applied to these matrices to calculate the integration of OFC with different networks, which quantifies the probability that two ROIs from different networks are assigned to the same community over time; higher values indicate greater cross-network communication. These brain-level metrics were then integrated with symptom, cognitive, and transcriptomic data for statistical analysis.

All subjects received a series of measurements including clinical symptoms, cognitive functions, MRI scanning, and EEG recording at the baseline and 4 weeks time session, with an additional clinical symptom assessment at 2 weeks (Fig. 1A). Clinical symptoms were evaluated by the positive and negative Syndrome scale (PANSS)^29^, global assessment function (GAF), clinical global impression (CGI), the Hamilton Anxiety Scale (HAMA), and Hamilton Depression Scale (HAMD). Cognitive function was measured by the MATRICS Consensus Cognitive Battery (MCCB)^30,31^. Detailed information on inclusion, exclusion criteria, randomization, blinding, and treatment protocol is provided in the “Methods” section and the previous study^28^.

### Clinical symptoms and cognitive function

We first examined the clinical effectiveness of rTMS. Symptoms and cognitive changes were reported in detail in Table 1 and the previous study^28^. In summary, we found that after 4 weeks of rTMS treatment, both total PANSS score gradually decreased and cognitive function improved in a time-dependent manner, exhibiting a significant group × session interaction (PANSS: *F*(2, 164) = 10.10, *p* < 0.001; MCCB: *F*(2, 164) = 11.60, *p* < 0.001). Compared to the sham group, symptom score was significantly reduced, and cognitive score was improved in the active group at 4 weeks (PANSS: *t* (82) = −3.96, *p* < 0.001; MCCB: *t* (82) = 2.33, *p* = 0.022). Furthermore, a similar group × session interaction was observed in the PANSS subscales, including negative symptom (*F*(2, 164) = 8.04, *p* < 0.001), positive symptom (*F*(2, 164) = 4.35, *p* = 0.02), and depressed symptom (*F* (2, 164) = 4.10, *p* = 0.02). The post-hoc analysis revealed that, compared to the sham group, these symptoms significantly decreased in the active group at 4 weeks (negative symptom: *t* (82) = −3.59, *p* = 0.003; positive symptom: *t* (82) = −2.13, *p* = 0.049; depressed symptom: *t* (82) = −2.64, *p* = 0.047).

**Table 1:** Demographic information, clinical symptoms, and cognitive function scores

	Baseline				2 Week				4 Week					
	Mean	SD	Mean	SD	Mean	SD	Mean	SD	Mean	SD	Mean	SD		
	Active (*N* = 45)		Sham (*N* = 39)		active		sham		active		sham		*F/Chi*	*p*
Age	26.91	8.39	26.73	7.90	-	-	-	-	-	-	-	-	0.10	0.920
Sex (F/M)	20/25		21/18	-	-	-	-	-	-	-	-	-	0.61	0.435
PANSSF1	26.82	5.30	26.41	5.76	23.22	5.08	24.13	6.43	18.98	4.30	21.87	7.04	**8.04**	**<0.001**
PANSSF2	16.64	3.93	17.51	3.14	14.42	3.12	15.95	3.24	12.89	2.60	15.13	2.98	2.45	0.089
PANSSF3	15.67	3.00	15.08	3.53	12.38	3.40	12.23	4.11	9.22	3.05	10.49	4.57	**4.35**	**0.014**
PANSSF4	6.51	2.60	6.13	2.18	5.53	2.31	6.00	2.56	4.76	1.84	5.46	1.77	2.11	0.125
PANSSF5	15.87	3.78	15.21	3.12	13.58	3.05	13.79	2.89	12.09	2.59	13.18	2.71	**4.10**	**0.018**
total	81.51	11.61	80.33	9.91	69.13	11.56	72.10	14.16	57.93	10.39	66.13	15.11	**10.10**	**<0.001**
CGI	4.53	0.55	4.54	0.68	3.98	0.54	4.05	0.76	3.40	0.65	3.77	0.90	**3.21**	**0.049**
GAF	34.80	6.52	37.08	7.64	42.76	6.85	41.74	9.44	51.64	8.15	46.72	11.73	**8.37**	**<0.001**
HAMA	12.40	3.20	11.85	3.75	9.62	2.81	9.38	3.00	7.22	2.80	8.08	3.06	2.19	0.115
HAMD	18.98	5.17	18.36	4.91	13.76	4.39	14.77	10.22	10.29	3.51	11.87	4.69	1.47	0.234
TMT	43.27	13.21	41.79	13.26	-	-	-	-	49.88	10.03	48.66	13.76	0.01	0.908
BACSSC	45.31	7.35	43.56	8.15	-	-	-	-	47.13	6.87	44.67	8.19	0.34	0.560
Fluency	50.98	9.49	46.56	14.20	-	-	-	-	50.42	11.48	46.74	14.69	0.09	0.762
AV	43.47	8.18	42.03	9.22	-	-	-	-	43.56	15.34	40.77	13.12	0.28	0.596
WM	44.20	12.24	43.67	12.75	-	-	-	-	47.51	13.30	44.49	16.04	0.76	0.384
VrbLrng	45.87	12.13	45.08	9.89	-	-	-	-	47.33	10.62	43.23	9.10	2.29	0.134
VisLrng	46.20	11.66	46.23	10.42	-	-	-	-	49.00	9.80	47.56	10.79	0.59	0.446
RPS	46.31	10.27	46.26	10.92	-	-	-	-	49.38	9.36	49.67	9.98	0.04	0.849
SC	34.22	6.21	33.23	5.02	-	-	-	-	34.76	6.68	32.62	6.15	0.79	0.374
total	42.26	7.31	42.10	8.84					46.90	6.54	42.62	9.58	**11.60**	**<0.001**

PANSSF1, PANSSF2, PANSSF3, PANSSF4, PANSSF5: Positive and Negative Syndrome Scale: Negative, Cognitive/Disorganization, Positive, Excitement, Depression/Anxiety.

*CGI* clinical global impression, *GAF* global assessment function, *HAMA* Hamilton anxiety scale, *HAMD* Hamilton depression scale, *TMT* trial making test, *BACSSC* brief assessment of cognition in schizophrenia: symbol coding, *AV* attention/vigilance, *WM* working memory, *VrbLrng* verbal learning, *VisLrng* visual learning, *RPS* reasoning and problem-solving, *SC* social cognition, *SD* standard deviation.

### Brain dynamic network integration

We next asked which brain downstream networks were perturbed by the OFC after rTMS intervention. This perturbation was measured by the dynamic integration coefficient, which describes the average probability that a given node *i* is in the same community as nodes from other networks over time (see Eqs. 1, 2 and “Methods” section). Conceptually, a node with high integration exhibits greater communication with nodes from other networks across the time windows^32,33^.

**Fig. 2:**
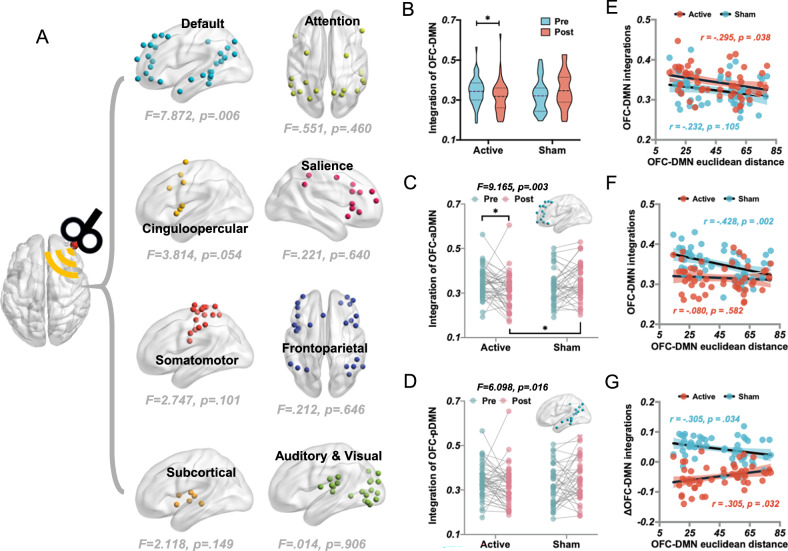
Low-frequency rTMS reduces dynamic integration between OFC and the default mode network. **A** Statistical results for the group × session interaction for integration between the OFC and eight canonical brain networks. Integration refers to “the average probability that the OFC is in the same community as nodes from each network across time windows”. Higher values indicate greater cross-network communication. A two-sided mixed-design ANOVA with factors group and session was conducted. A significant interaction was observed only for the Default Mode Network (DMN) (F(, 82) = 7.872, partial η² = 0.09, Bonferroni-adjusted *p* = 0.048). Eight distinct colors denote eight brain networks. Violon plots show the changes in integration coefficients between OFC and the whole DMN (**B**), the anterior DMN (aDMN; **C**), and the posterior DMN (pDMN; **D**). Data are presented as mean values ± SEM. Blue violin represents the values of pre-intervention, and red violin represents the values of post-intervention. Dashed lines represent the median and quartiles. Gray lines connect data points from the same subject, illustrating individual changes between pre-intervention and post-intervention. **E**–**G** Scatter plots showing the relationship between OFC-DMN integration and Euclidean distance. Euclidean distance is the physical distance in MNI space (mm) between each DMN ROI and the OFC stimulation target. Lines represent linear regression fits, and shaded areas represent the 95% confidence intervals of the regression line around the mean predicted value. Pearson correlations were calculated using two-sided tests. This analysis tests the hypothesis that rTMS effects are spatially constrained. **E** At baseline, a negative correlation exists, indicating that more distant ROIs are less integrated with OFC. **F** After treatment, this relationship is abolished in the active group. **G** The change in integration (ΔOFC-DMN) is positively correlated with distance in the active group, indicating that the greatest reduction in integration occurred in ROIs closest to the stimulation target. Red dots represent values of the active group, blue dots represent values of the sham group, and shaded areas represent 95% confidence intervals (95% CI). In all panels, * denotes *p* < 0.05, uncorrected. Source data are provided as a Source Data file.

As shown in Fig. 1B, we defined the whole-brain using spherical ROIs and calculated the integration coefficients between the right OFC target and the eight functional networks in the Power 264 atlas, including the Somatomotor network (SMN), Cingulo-opercular network (CON), Audio-Visual network (AVN), Default mode network (DMN), Fronto-parietal network (FPN), Salience network (SN), Subcortical network, and Attention network (AN). A mixed-design ANOVA analysis showed that the interaction between group and session on integration between right OFC and DMN was significant (Fig. 2A), F(1,82) = 7.872, *p*_*Bonferroni*_ = 0.048, *partial η*² = 0.09. Post-hoc analysis found that, after 4 weeks of rTMS treatment (Fig. 2B), integration between the right OFC and DMN was significantly reduced in the active group (*t*(82) = 2.15, *p* = 0.035, *Cohen’s d* = 0.24), whereas no such effect was observed in the sham group (*t*(82) = −1.83, *p* = 0.070, *Cohen’s d* = 0.20). To further explore the specificity of this effect, we subdivided the DMN into anterior DMN and posterior DMN (Fig. 2C, D). We found that anterior DMN exhibited a stronger treatment response (*F*(1,82) = 9.165, *p* = 0.003, *partial η*² = 0.10). Integration between the OFC and anterior DMN was significantly decreased in the active group after rTMS treatment (*t*(82) = 2.57, *p* = 0.012, *Cohen’s d* = 0.28), whereas no such effect was observed in the sham group (*t*(82) = −1.75, *p* = 0.084, *Cohen’s d* = 0.19). We also conducted a left–right hemispheric division but found no evidence of right-sided dominance. Significant group differences were observed in both hemispheres (left: *F*(1,82) = 7.544, *p* = 0.007, *partial η*² = 0.08; right: *F*(1,82) = 6.636, *p* = 0.012, *partial η*² = 0.07; see Supplementary Fig. S1).

The stronger response of the anterior DMN to treatment suggests that OFC-induced physiological effects have diminishing marginal effects, such that their influence decreases with increasing brain distance. To test this hypothesis, we obtained group-level integration coefficients between the DMN’s 50 ROIs and the OFC, as well as their Euclidean distances from the OFC. The distance was defined as the linear physical distance (in millimeters) between each DMN ROI and the center coordinates of the right OFC stimulation target in the MNI space. Pearson correlation analysis revealed that, at baseline, integration between the OFC and DMN ROIs decreased as Euclidean distance increased in both the active (*r* = −0.295, *p* = 0.038) and sham (*r* = −0.232, *p* = 0.105) group (Fig. 2E). However, this marginal diminishing effect was reversed in the active group (*r* = −0.080, *p* = 0.582) after rTMS intervention (Fig. 2F). To further characterize the directional changes in integration coefficient induced by OFC stimulation, we further calculated the correlation between the difference of integration (post-pre/post+pre) and the distance. The active group showed a significant positive correlation (*r* = 0.305, *p* = 0.032; Fig. 2G), indicating that the closer the physical distance between DMN’s ROIs and OFC was, the lower their integration value would be after the intervention. We also compared the correlations of distance-dependent effects between the two groups at the individual level. A two-sample *t*-test showed marginal significance (active > sham, *t* = 1.944, *p* = 0.055, see supplementary Fig. S2). Although it did not reach significance, combined with the group-level correlation findings, this to some extent indicates that OFC-induced physiological effects indeed reduce integration with the DMN in SCZ, and such intervention effect decreases with increasing brain distance.

### Decompose the integration pattern using non-negative matrix factorization

**Fig. 3:**
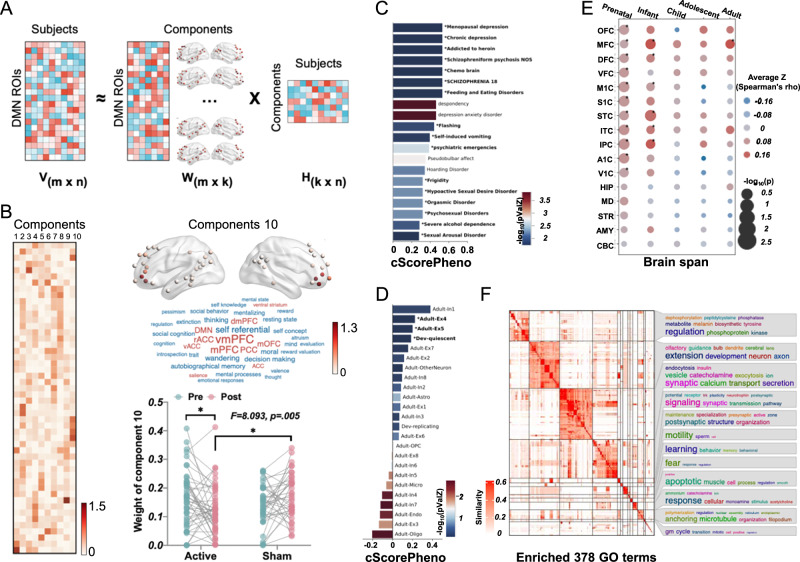
A vmPFC-dominated integration pattern is modulated by rTMS and is spatially correlated with schizophrenia-related gene expression. **A** Schematic of non-negative matrix factorization (NMF), a data-driven method used to decompose the subject-by-ROI integration matrix into a set of spatial components (W) and their corresponding subject-level weights (H). **B** NMF identified 10 components. A two-sided mixed-design ANOVA with factors group (active, sham) and session (pre, post) revealed a significant group × session interaction for Component 10, spatially dominated by the ventromedial prefrontal cortex (vmPFC) (F(1,82) = 8.093, partial η² = 0.09, Bonferroni-adjusted *P* = 0.005). The bar chart shows the weights for all 10 components, and the line plot illustrates the significant post-treatment reduction in the weight of component 10 in the active group compared to the sham group. Word clouds represent cognitive terms associated with Component 10. **C**–**F** Exploratory analysis showing the spatial correlation between the Component 10 map and various gene expression profiles from publicly available databases. This analysis assesses the biological plausibility of the component. **C** The component’s topography is correlated with genes associated with schizophrenia-like psychosis. **D** It is enriched for excitatory neuronal cell-type markers (Ex4, Ex5). **E** Gene expression patterns are associated with developmental stages of the OFC and related regions. **F** Genes are enriched in biological processes related to synaptic function and signal transduction. In all panels, * denotes *p* < 0.05, uncorrected. Source data are provided as a Source Data file.

To further identify key regions affected by rTMS treatment, we used the non-negative matrix factorization (NMF; Eq. 3) method to identify ROI level sub-graphs nested within the network-level pattern. NMF constrains all values in the decomposition to be exclusively positive. Compared to common methods like independent component analysis (ICA), NMF provides more interpretable, consistent, and precise results. Here, for all subjects, the integration coefficients between the OFC and 50 nodes within the DMN at two time points (pre-session and post-session) were used as the inputs for NMF (Fig. 3A). As a result, NMF identified 10 distinct components representing integration sub-graphs between the right OFC and DMN (supplementary Fig. S3). Notably, the 10th component dominated by the ventromedial prefrontal cortex (vmPFC; Fig. 3B) exhibited a significant group × session interaction (*F*(1, 82) = 8.09, *p*_*Bonferroni*_ = 0.05, *partial η*² = 0.09). Post-hoc analysis revealed a significant reduction in integration within the active group following rTMS treatment (*t*(82) = 2.32, *p* = 0.023, *Cohen’s d* = 0.26), whereas no significant change was observed in the sham group (*t*(82) = −1.73, *p* = 0.087, *Cohen’s d* = 0.19). At the ROI level, the prefrontal region of the DMN, particularly the vmPFC, appeared to be a key target affected by OFC stimulation.

### Genetic associations with brain network dynamics

The etiological basis of schizophrenia is the combination of multidimensional factors, involving environment, neurology, and genetics^2^. Uncovering the mapping relationship between the brain dynamic network and genetics could allow for disease-related biological pathway identification. To investigate the biological plausibility of this rTMS-sensitive integration pattern, we performed an exploratory analysis correlating its spatial topography with transcriptional maps from the Allen Human Brain Atlas. The goal was to determine if the brain regions most modulated by the treatment were enriched for genes known to be relevant to schizophrenia pathophysiology. We examined the correlation between integration coefficients across 50 ROIs within the DMN and gene expression profiles from the Allen Brain Atlas, resulting in a series of significant genotypes, which were further decomposed into neuronal cell-type markers (PsychENCODE), genes associated with psychiatric disorders (DisGeNET), developmental region gene enrichment (BrainSpan), and gene function (Gene Ontology, GO) biological process associations. We found that genes spatially associated with changes in integration within the NMF10 component were significantly linked to schizophrenia-like psychosis and showed high comorbidity with schizophrenia (Fig. 3C). Specifically, these genes were enriched in excitatory neuronal subtypes, including Ex4 and Ex5, and Dev-quiescent neurons (Fig. 3D), which are related to schizophrenia pathophysiology. It was also involved in the developmental stages of OFC related region (Fig. 3E). Further semantic clustering analysis based on GO categories showed that genes spatially associated with integration coefficient in NMF10 were enriched in biological processes related to synaptic function, signal transduction, and learning (Fig. 3F). Together, these findings suggest that the NMF10 component, a key integration pattern modulated by rTMS, is spatially aligned with a molecular landscape implicated in schizophrenia. This correlation provides convergent, cross-scale evidence for the biological relevance of our neuroimaging findings, though it does not imply a causal relationship.

### Exploring the relationship among brain measurements, clinical symptoms, and cognitive functions

**Fig. 4:**
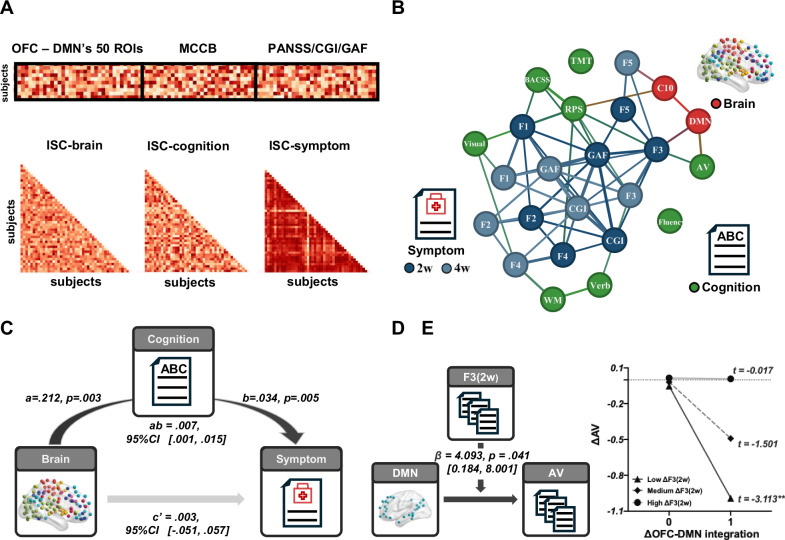
Cognitive function mediates, and positive symptoms moderate the effects of OFC-DMN modularity. **A** Inter-subject correlation (ISC) analysis. ISC matrices show the similarity between every pair of subjects (active group) based on their treatment-induced changes in brain integration, cognitive performance, and clinical symptoms. Higher values (warmer colors) indicate greater similarity. **B** Mediation analysis results. A linear regression-based mediation model was implemented with bootstrap resampling. The indirect effect (path a × b) was significant (β = 0.007, 95% CI [0.001, 0.015]). The direct effect (path c′) was not significant (β = 0.003, 95% CI [ − 0.051, 0.057]). Full mediation was inferred because the confidence interval of the indirect effect did not include zero. All tests were two-sided. **C** Correlation network of specific sub-domains. The graph illustrates significant correlations (*p* < 0.05, uncorrected) between treatment-induced changes in specific brain (red), cognitive (green), and symptom (blue) measures. Line thickness corresponds to the strength of the correlation. **D** Moderation analysis model. The model tests whether the effect of OFC-DMN integration changes on attention/vigilance (AV) is conditional upon the level of positive symptom change (F3 at 2 weeks). A linear regression model including the interaction term was used. **E** Simple slope plot of the moderation effect. The relationship between reduced OFC-DMN integration (more modularity) and improved AV performance is significant only when there is a reduction in positive symptoms (low ΔF3(2w), solid line). Statistical significance is indicated as **p < 0.01, uncorrected. PANSS: positive and negative syndrome scale. F1 negative factor. F2 disorganized factor. F3 positive factor. F4 excited factor. F5 depressed factor. CGI clinical global impression. GAF global assessment function. TMT trail making test. BACSS brief assessment of cognition, schizophrenia: symbol coding. AV attention/vigilance. WM working memory. RPS reasoning and problem-solving. Source data are provided as a Source Data file.

So far, the effectiveness of 1 Hz rTMS targeting the right OFC in people with schizophrenia has been revealed through three levels: clinical symptoms, cognitive function, and brain network alterations. However, an important question remains: how do these factors interact and co-vary in response to rTMS? To address this, we performed an inter-subject correlation (ISC) analysis to examine covariation among brain integration, cognitive function, and clinical symptoms in the active group (Fig. 4A). For each subject, intervention differences in the neural, symptom, and cognitive sub-dimensions were obtained and concatenated. We then calculated the similarities between the subjects in these sub-dimensions to form an ISC matrix at three different levels. The results revealed a significant positive correlation between neural ISC and cognitive ISC (*r* = 0.093, *p* = 0.003), as well as between symptom ISC and cognitive ISC (*r* = 0.090, *p* = 0.004). These findings suggest a degree of synchronization among the three domains, with cognition appearing to serve as a bridge between neural and symptom changes. Subsequent mediation analysis confirmed this, showing that cognitive function has a mediation effect on the integration change rate of clinical symptoms. The coefficient of the indirect effect (path a × b) was significant (*β* = 0.007, 95% CI: [0.001, 0.015]). The direct effect (path c′) was not significant (*β* = 0.003, 95% CI: [−0.051, 0.057]; Fig. 4B). Thus, at the global level, brain intervention response predicted symptom improvement and was fully mediated by cognitive function.

A key remaining question is: Do OFC-induced physiological effects improve specific symptoms and cognitive factors? To screen for specific factors, we calculated the correlations between intervention differences in all sub-dimensions at the three levels. Significant correlations among these sub-domains are depicted in Fig. 4C. Notably, we found that OFC-DMN integration, positive symptoms (F3) at two weeks, and attention/vigilance (AV) scores were interrelated. Subsequent modulation analysis showed that positive symptoms significantly moderated the effect of OFC-DMN integration difference on AV difference (*β* = 4.093*, p* = 0.041, 95% CI: [0.184, 8.001]; Fig. 4D). Simple slope analysis showed that only when the positive symptoms decreased, the reduced integration would significantly improve the AV cognitive function (low △F3: *t* = −3.113, *p* = 0.003; medium: *t* = −1.501, *p* = 0.138; high: *t* = −0.017, *p* = 0.984; Fig. 4E). These findings provide further evidence that OFC-induced neural changes lead to a decoupling of communication with downstream DMN, and such modularity improves subjects’ attentional alertness through moderation of positive symptoms.

### Exploratory analysis of attentional alertness

Attentional alertness may reflect abnormal salience processing in positive symptoms^34^, and tends to be more connected to the attention network than the DMN^35,36^. One potential explanation is that the DMN may act as a relay, transmitting the intervention effect to the downstream attention network. To explore this specific, network-level hypothesis, we employed Granger causality analysis in an exploratory capacity. Critically, our aim was not to infer causality between brain regions, but rather to test whether dynamic fluctuations in the upstream network coupling (OFC-DMN integration) causally influenced the fluctuations in downstream network coupling (DMN-AN integration). Accordingly, we used the time series of the integration coefficients for these two network pairs as inputs for the Granger causality analysis.

**Fig. 5:**
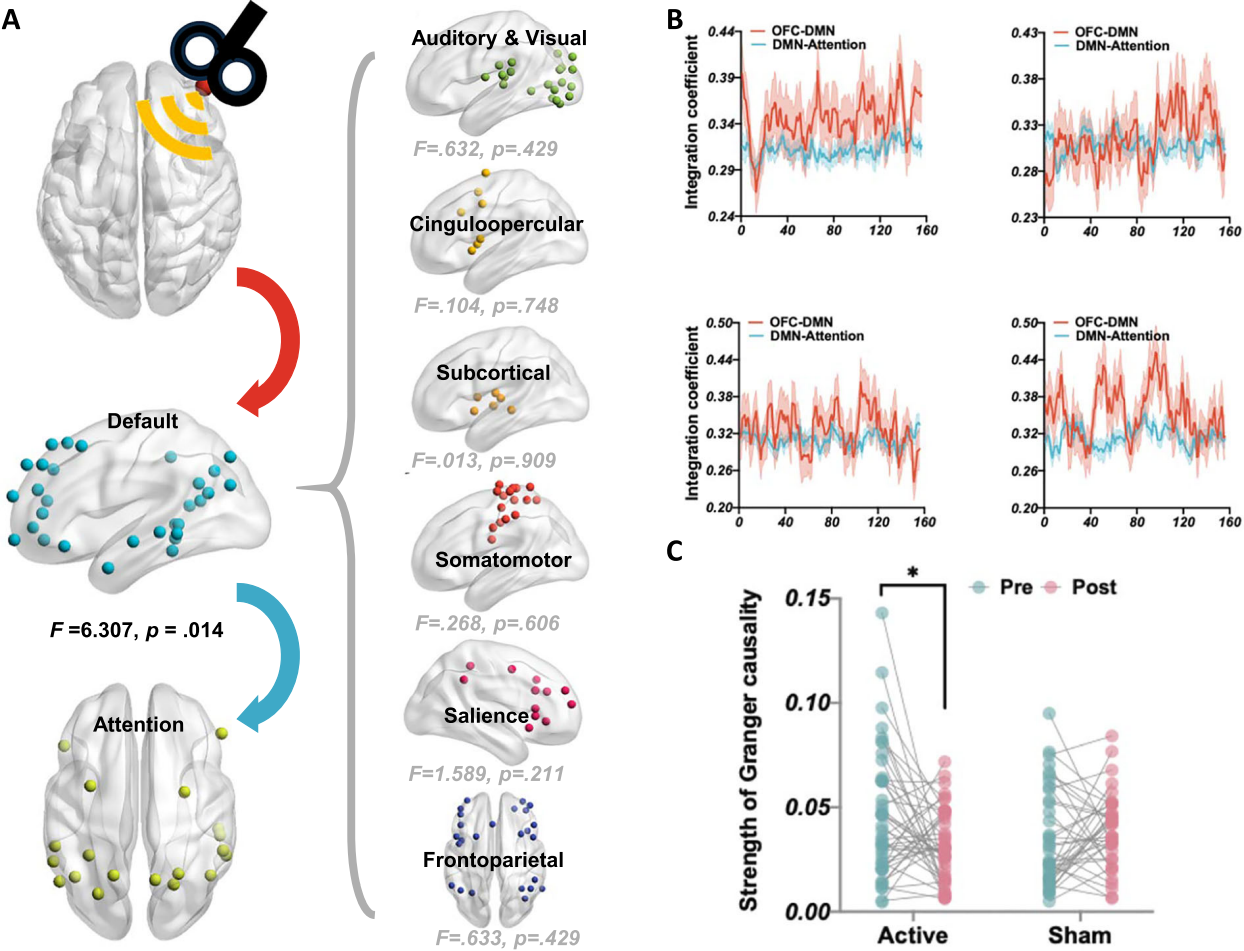
rTMS attenuates the causal influence from the pathway OFC-DMN-attention network. **A** Granger causality (GC) was used to test the hypothesis that dynamic changes in OFC-DMN coupling causally influence downstream DMN-Attention Network (AN) coupling. A two-sided mixed-design ANOVA with factors group (active, sham) and session (pre, post) was used. Among the seven downstream networks, significant interactions were only observed in the DMN-AN pathway (F(1,82) = 6.307, partial η² = 0.07, *P* = 0.014). **B** Group-average time series of integration coefficients for the OFC-DMN (red) and DMN-AN (blue) pathways, before and after the intervention for both active (top) and sham (bottom) groups. Shaded areas represent the standard error of the mean. **C** Line plot showing the strength of GC from the OFC-DMN to the DMN-AN pathway. A two-sided mixed-design ANOVA with factors group (active, sham) and session (pre, post) was used. Post-hoc two-sided paired *t*-tests show that the active group shows a significant post-treatment reduction in causal influence, an effect not observed in the sham group. Individual gray lines connect data points from the same subject across sessions. In all panels, * denotes *p* < 0.05, uncorrected. Source data are provided as a Source Data file.

Mixed-design ANOVA analysis showed a significant group × session interaction in causality across OFC-DMN-AN (*F* = 6.307, *p* = 0.014, *partial η*² = 0.07; Fig. 5A). Figure 5B shows the group-level time series before and after the intervention. Notably, no significant causal interactions were observed between OFC-DMN time series and DMN-other time series, suggesting that this causal influence is specific to the AN. Post-hoc analysis showed that the causal influence of OFC-DMN on DMN-AN was decreased in the active group after intervention (*t* = 2.488, *p* = 0.017, *Cohen’s d* = 0.37; Fig. 5C), whereas this effect was not observed in the sham group. In addition, we observed that the reduced causal influence may be affected by the extreme values of the active group in the baseline. We therefore excluded the two subjects in the active group whose pretest causal influence exceeded 0.1 and repeated the analysis with the remaining subjects. The results still showed a significant interaction (*F* = 4.352*, p* = 0.040, *partial η*² = 0.05), and the reduction of Granger causality in the active group was close to significant (*t* = 1.950*, p* = 0.058, *Cohen’s d* = 0.29, see supplementary Fig. S4). After accounting for potential regression to the mean, our analysis provides preliminary evidence suggesting that the rTMS-induced modularity between the OFC and DMN may attenuate the DMN’s causal influence on the downstream AN. Although this attenuation is consistent with the inhibitory effects of 1-Hz rTMS and our overall hypothesis, we should interpret this finding with caution: this could also reflect the disruption of a potentially important regulatory mechanism, a possibility that our data cannot exclude.

## Discussion

In this study, through an RCT investigating 1 Hz rTMS targeting the right OFC in SCZ, we assessed the dynamic influence of OFC-induced physiological effects on downstream brain networks. The OFC selectively reduced integration with the DMN following active rTMS intervention, and such an effect was modulated by brain distance. This could be interpreted as reduced communication between DMN and OFC, leading to modularity. Similarly, this effect was further transmitted downstream, making AN, as an antagonistic network of DMN, less perturbed, which may be the basis for improving symptoms and cognition. Specifically, within the DMN, we observed that the reduction in integration was particularly concentrated in a vmPFC-dominated pattern, which was also linked to schizophrenia-related gene expression. We subsequently revealed that global neural, cognitive, and symptomatic changes were synchronized across subjects. Encouraged by this, we further focused on specific intervention pathways and found that alterations in brain integration selectively improved attentional performance, with this improvement being moderated by positive symptoms. Exploratory Granger causality analysis provided preliminary evidence for a potential mechanism: the causal influence from the OFC-DMN pathway to the downstream DMN-AN pathway was reduced in the active group. While requiring cautious interpretation, these findings hint that rTMS may work by decoupling the DMN from its downstream antagonistic networks. Taken together, low-frequency rTMS over the right OFC may reduce its integration with the DMN, which in turn transmits this effect to the downstream AN. Crucially, this neural change improved the SCZ’s positive symptoms, especially attentional alertness.

### OFC-induced physiological effects selectively decrease dynamic integration with the DMN in schizophrenia

A major finding of this study is that 4 weeks of low-frequency rTMS treatment significantly reduced the dynamic integration between the OFC and DMN in people with schizophrenia. These findings are encouraging as extensive literature has documented the DMN to be implicated in spontaneous cognitive activity and self-referential processes^6,37,38^. Its hyperactivation and hyperconnectivity may be considered hallmark features of schizophrenia, contributing to the observed reduced modularity of network^39^. A study in people at ultra-high risk for psychosis examined resting-state connectivity of the salience network (SN) and DMN in association with facial emotion recognition^40^. They report increased connectivity between the SN and the medial prefrontal cortex, an area of the DMN, in participants at ultra-high risk for psychosis. Our results show that the putatively normalizing effect of rTMS was particularly pronounced in a component dominated by the vmPFC, a core hub of the DMN. Anatomically, the OFC relays multi-sensory information to downstream structures via the vmPFC, playing a crucial role in regulating emotion and motivation^22,41^. Therefore, the excessive integration between the OFC and DMN observed in psychosis-risk states may blur the boundary between internal thoughts and external reality. This disruption leads to an exaggerated focus on self-referential thoughts, where neutral events are imbued with personal significance—a mechanism believed to underpin core symptoms like paranoid ideation and hallucinations^6,42^. By reducing this aberrant integration, our rTMS protocol may help restore a more balanced and modular network architecture.

An alternative, yet complementary, interpretation of our findings on OFC-DMN connectivity relates to the well-established role of the right OFC in processing non-reward signals^43^. This theory posits that the OFC contains specialized neurons that, in response to non-reward or punishment, can enter a persistent “attractor state” maintaining their firing for an extended period. This sustained activity acts as a memory of the negative event. In this context, excessive integration between the OFC and the DMN could signify an over-representation of negative emotional and motivational information within the self-referential DMN. This could lead to a persistent, negative, and self-focused bias that contributes significantly to the clinical experience of negative symptoms. From this perspective, the observed therapeutic effect in our study, the improvement of negative symptoms, would be a result of the intervention helping to uncouple this pathological non-reward activity from the DMN, thereby alleviating the persistent negative bias and its associated symptoms.

### The vmPFC-dominated integration pattern is supported by the schizophrenia-related gene phenotype

Furthermore, our exploratory analysis of gene expression patterns adds biological plausibility to this network-level finding. The spatial map of the vmPFC-dominated integration pattern was significantly correlated with the expression of genes linked to schizophrenia risk. Specifically, this map was enriched for genes related to excitatory neurons, synaptic function, and signal transduction^44,45^. This spatial association does not imply that these genes mechanistically drive the treatment response, but rather provides important, cross-scale validation. The large-scale network dynamics modulated by our rTMS protocol are rooted in the specific molecular and cellular systems long implicated in the pathophysiology of schizophrenia. As previous research pointed out, brain networks may be viewed as ‘intermediate phenotypes’, mediating the causal pathway from genetic variation to behavioral manifestations^46,47^. In this context, our findings suggest that OFC-targeted rTMS may provide a precise therapeutic intervention for schizophrenia, mediated by the identified biomarker—network integration patterns that drive cognitive improvements and symptom reduction. These results further support the utility of imaging connectomics as a powerful framework for elucidating how genetic risk factors for psychiatric disorders manifest through alterations in large-scale brain networks.

### Positive symptoms moderate the influence of OFC-DMN communication on attention/vigilance

Another important finding is that OFC-induced treatment effects on brain dynamic integration, cognitive functions, and clinical symptoms exhibited a cross-subject correlation. Stimulation reduced the integration between the OFC-DMN network, and this alteration further facilitated cognitive improvement, along with symptom alleviation. From the modulation model, we observed that the reduction of integration specifically predicted improvements in attention performance, with this relationship being moderated by changes in positive symptoms. The neural basis of this relationship was validated through Granger causality analysis, which demonstrated a significantly reduced causal influence of OFC-DMN on DMN-Attention Network (DMN-AN) connectivity following active rTMS treatment. This finding is particularly relevant given that cognitive impairments represent a core feature of schizophrenia, where DMN hyperactivation and hyperconnectivity contribute to both cognitive deficits, particularly in attention and working memory, and positive symptoms^42,48,49^. Recently, a working model of neural activity and phenomenal experience proposed that DMN hyperactivity (the “associative unit” in the model) may drive aberrant top-down signals to sensory cortices, leading to a dissociation of phenomenal experience from the external environment, which manifests as positive symptoms like hallucinations and delusions^50^. From this perspective, the reduced OFC-DMN integration observed following our rTMS intervention may not represent a simple suppression of function, but rather a therapeutic normalization of this pathological DMN hyperactivity. By modulating an anterior hub of the DMN, the intervention may dampen this excessive top-down “associative drive”. This rebalancing of large-scale network dynamics would, in turn, restore a healthier state of segregation between the internally-focused DMN and the externally-focused AN. The subsequent reduction of DMN’s influence over the AN could free up crucial cognitive resources, allowing for improved attentional control and a diminished intrusion of the aberrant internal experiences that constitute positive symptoms. The exploratory Granger causality finding of the OFC-DMN-AN pathway provides preliminary support for this proposed mechanism.

### Implications and strengths

Schizophrenia is increasingly recognized as a heterogeneous disorder, both clinically and genetically. The present findings, using multiple methods, highlight the right OFC as a potential precise intervention localization specific for addressing positive symptoms and attention deficit in schizophrenia. The observed distance-dependent effects provide evidence that our stimulation protocol engaged the intended OFC-centered circuitry. However, it is important to acknowledge that, in the absence of comparison with other stimulation sites, the extent to which the observed clinical effects are unique to OFC stimulation remains an important open question. Besides, the significant improvement observed in the PANSS depressed subscale, despite the lack of change in the broader Hamilton scales, suggests that specific emotional symptoms that are associated with schizophrenia psychopathology may improve alongside improvements in other symptoms. This highlights the potential of our intervention to address the complex emotional dysregulation often present in this population. By integrating multidisciplinary approaches—spanning molecular, network, and behavioral levels of analysis—tailored interventions can be developed to address specific impairments, thereby advancing the realization of precision medicine in psychiatric disorders.

### Limitations

This study has several limitations. First, our study lacks a healthy control group. Without establishing baseline network integration levels in a non-clinical population, we cannot definitively conclude that the observed reduction in OFC-DMN integration represents a normalization of pathologically excessive connectivity. Future studies including a healthy control group would be crucial to confirm this interpretation. Second, although we observed an improvement in overall cognitive scores, no specific cognitive domain showed distinct enhancements, limiting the scope of conclusions about specific cognitive benefits. Third, our inferences about OFC treatment specificity are also limited, given the absence of other stimulation targets for comparison. Furthermore, the lack of long-term follow-up means the stability of these improvements remains an important question that needs to be answered in future studies. Fourth, the limited temporal resolution afforded by 156 time windows may be insufficient for robust causality inference. These Granger causalities should be viewed as preliminary, exploratory findings. Finally, discrepancies existed between the mediation and moderation analyses, which warrant careful interpretation. While these findings might seem to represent contradictory causal models, we speculate that they may not be mutually exclusive: they may capture phenomena at different analytical levels. The mediation model, using global scores, may reflect a macro-level pathway where broad cognitive improvements contribute to overall symptom reduction. The moderation model, using specific variables, could reveal micro-level dynamics, where the translation of specific neural gains into improved attention is conditional upon the patient’s positive symptom state. Under this hypothetical framework, it is possible that while cognitive enhancement is a primary pathway to recovery (mediation), the efficiency of this pathway for certain domains is dynamically regulated by concurrent symptoms (moderation). These exploratory analyses underscore the complex, likely bidirectional interplay between cognitive and symptomatic changes following rTMS. We propose this not as a definitive conclusion, but as a testable hypothesis for future research. Potential sources of bias should be acknowledged. Recruitment from a single psychiatric hospital may limit the generalizability of the findings. In addition, participation required a willingness to undergo rTMS treatment and provide informed consent, which may have led to underrepresentation of patients with poor insight, severe agitation, or lower treatment adherence. These factors should be considered when interpreting the applicability of the results to broader schizophrenia populations.

In conclusion, we employed low-frequency rTMS to stimulate the right OFC, which effectively reduced the dynamic network integration between OFC-DMN, reduced clinical symptoms, and improved cognitive function associated with schizophrenia. We suggest that less interference from the DMN to the attention network may be beneficial for people with schizophrenia. Of interest, these neural changes were associated with schizophrenia-related gene expression, emphasizing the interplay between genetic, neural, and clinical factors. Importantly, these results underscore the role of heterogeneous neural systems in SCZ and suggest that targeted rTMS can be a promising tool to modulate brain modularity and improve clinical outcomes.

## Methods

### Participants

Participants were consecutively recruited from the inpatient and outpatient departments of the Harbin First Specialized Hospital between November 2019 and December 2021. Potentially eligible patients were screened by trained psychiatrists according to DSM-IV criteria. All eligible patients who met the inclusion criteria during the recruitment period were invited to participate. The inclusion criteria included: (1) diagnosed with schizophrenia according to the DSM-IV diagnostic criteria, (2) willing to receive rTMS therapy and provide signed informed consent, (3) 15–45 years old and IQ > 69, (4) drug-naïve and experiencing their first episode of psychosis, (5) positive and negative syndrome scale (PANSS) score ≥60, and (6) overall clinical global impression (CGI) scale ≥4. The subjects received atypical antipsychotics after enrollment. The exclusion criteria included: (1) sensorimotor disorders, neurological diseases, or other physical diseases, (2) rTMS treatment contraindications such as metal implants, obvious excitement, and irritability, (3) having received ECT treatment within one month, and (4) pregnancy. An independent third party sorted subjects into either the real or sham groups via computer-generated randomization.

The study was approved by the Ethics Committee of Shanghai Mental Health Center and The First Psychiatric Hospital of Harbin and registered with the Chinese Clinical Trial Register Center (Registration number: ChiCTR2000041106). All subjects provided informed consent.

### Experimental design

In this RCT study, we conducted a 4-week rTMS intervention. Subjects were randomly assigned to the active rTMS or sham rTMS group. An independent third party divided subjects into either the active or sham groups via computer-generated randomization. The clinical staff and subjects were blind to the assignment, except for one clinical technician who provided the rTMS or sham treatment according to the randomization numbers. During intervention, all subjects received a series of measurements, including clinical symptoms, cognitive functions, MRI scanning, and EEG recording at the baseline and 4 weeks, with an additional clinical symptom assessment at 2 weeks (Fig. 1A). In the current study, we analyzed the resting fMRI data separately.

### Stimulation protocol

The subjects were instructed to sit on a comfortable chair in a quiet room, and rTMS was delivered using the MagPro X100 magnetic stimulator (Medtronic Co., Denmark) equipped with a butterfly coil (cool d-B80). Similar to the previous study^19^, the intervention was administered using a MagPro ×100 stimulator equipped with a 120°-angled figured 9 coil (Cool D-B80) and a coil cooler unit (MagVenture). In the active rTMS group, the stimulation was targeted to the right orbital frontal cortex with the coil vertex positioned over the AF8 site, which was defined according to the international 10–10 EEG system. The coil was oriented with the handle vertical and upward, perpendicular to the axial plane of the head, and current flow directed superiorly, for optimal stimulation of the horizontal shelf of the OFC. Each rTMS procedure consisted of 12 trains of 60-s duration separated by 30-s inter-train intervals, for a total of 17 min and 30 s. The frequency of rTMS was 1 Hz, the intensity of rTMS was 110% resting motor threshold (RMT), and the total number of pulses was 720. The subjects received rTMS treatment for 20 consecutive days. The precision of right OFC stimulation was also evaluated via realistic calculations of the electric field through SimNIBS tools^51^, and can be found in supplementary Fig. S5. The same protocol and the same coil were used for the sham group, except that the coil was flipped 180°, so it produced the same “click” sound but was unable to produce a magnetic field. Besides, scalp electrodes were placed in both groups to record TMS–EEG activity. These electrodes were not used to mimic scalp sensations. The subjects were blinded to the treatment they received. An independent third party sorted participants into either the active or sham groups via computer-generated randomization. More design details and a part of clinical outcomes were also reported in our previous publications^25,28^.

### Assessment

#### Clinical symptoms

Clinical psychopathology was assessed using PANSS. The PANSS consists of 30 items, divided into five subscales: negative, disorganized, positive, excited, and depressed factors^29^. Each item was rated on a 7-point Likert scale (1 = absent to 7 = extreme). Clinical Global Impression (CGI) and global assessment of functioning (GAF) were used to assess the severity of the disease and the overall functioning of subjects. Anxiety and depression severity were measured by the Hamilton Anxiety Scale (HAMA) and Hamilton Depression Scale (HAMD).

#### Cognitive function

Cognitive function was measured by the MATRICS Consensus Cognitive Battery (MCCB). It was divided into several subscales^31^: trail making test (TMT), brief assessment of cognition schizophrenia: symbol coding (BACSSC), category fluency: animal naming (Fluency), attention/vigilance (AV), working memory (WM), verbal learning (VrblLrng), visual learning (VisLrng), and reasoning and problem-solving (RPS).

All clinical and cognitive assessments were conducted under standardized conditions in the same setting by the same senior psychiatrist, blinded to the subjects’ group allocation. PANSS, CGI, and GAF scores were assessed at baseline, week 2, and week 4, while MCCB scores were evaluated at baseline and week 4.

### Neuroimaging

#### Image acquisition

Imaging data were acquired on a GE Discovery MR750 3Tesla MRI scanner system with a 32-channel head coil. Anatomical images for registration to Montreal Neurological Institute (MNI) template space were acquired for each participant using a T1-weighted Magnetization acquisition with Spin Echo sequence: TR = 8.20 ms, TE = 3.22 ms, voxel size = 1 × 1 × 1 mm, flip angle = 12°, field of view (FOV) = 256 mm. Functional images were acquired using gradient-echo echo-planar imaging (EPI) with the following parameters: TR = 2000 ms, TE = 45 ms, FOV = 200 mm, thickness = 4 mm, slices = 32, flip angle = 90°, voxel size 2 × 2 × 2 mm.

#### Preprocessing

Both structural and functional MRI data were minimally preprocessed using fMRIPrep 23.1.4 based on Nipype 1.8.6. The processing steps for anatomical and functional MRI data are summarized below:

Anatomical data preprocessing. T1-weighted (T1w) structural images were corrected for intensity non-uniformity (INU) using N4BiasFieldCorrection^52^, implemented in ANTs^53^. Skull stripping was performed on the T1w-reference using the antsBrainExtraction, with the OASIS30ANTs template as the target for brain extraction. Following brain extraction, tissue segmentation was carried out on the extracted T1w image using the FSL FAST tool (https://www.win.ox.ac.uk/analysis/research/fast) to delineate cerebrospinal fluid (CSF), white matter (WM), and gray matter (GM) compartments. Spatial normalization was performed using nonlinear registration with the ANTs antsRegistration tool, aligning the brain-extracted T1w-reference to the MNI space (MNI152NLin6Asym).

Functional data preprocessing. The preprocessing of resting-state fMRI data (rs-fMRI) was performed as follows: First, a reference volume and its skull-stripped version were generated using a custom methodology from fMRIPrep. Head-motion parameters, including transformation matrices and six corresponding rotation and translation parameters, were estimated with respect to the rs-fMRI reference using mcflirt. Next, slice-timing correction was applied to the rs-fMRI using 3dTshift from AFNI. The data were resampled back onto native space by applying transforms to correct for head motion. The rs-fMRI reference was co-registered to the T1w-reference using mri_coreg (FreeSurfer), followed by FLIRT with a boundary-based registration cost function^54^. Co-registration was performed with six degrees of freedom. The rs-fMRI were then resampled into standard space, generating a preprocessed data in MNI152NLin6Asym space (resolution = 2 mm). To account for confounding factors, framewise displacement (FD), DVARS, and three region-wise global signals were calculated. Both FD and DVARS were calculated for each functional run using their implementations in Nipype. The three global signals were extracted from the cerebrospinal fluid (CSF), white matter (WM), and whole-brain masks. Additionally, physiological regressors were extracted to enable component-based noise correction (CompCor), including temporal (tCompCor) and anatomical (aCompCor) variants.

Physiological denoising. The denoising processing was performed by eXtensible Connectivity Pipelines (XCP-D) for mitigating motion artifacts and noise in rs-fMRI data. The first ten volumes were discarded as non-steady-state volumes. 36 nuisance parameters were regressed from time series with a general linear model, including six motion parameters, global signal, mean white matter signal, and mean CSF signal, along with their temporal derivatives, quadratic terms, and quadratic derivatives^55^. Time series intensity outliers were identified and interpolated with AFNI’s 3dDespike. Volumes with filtered FD greater than 0.5 mm were cubic spline interpolated in the BOLD data. Residual time series were band-pass filtered (second-order Butterworth) for retaining signals between 0.01 and 0.08 Hz. Finally, the denoised data was smoothed with a Gaussian kernel (FWHM = 6 mm).

#### Dynamic functional connectivity (dFC) matrix construction

The dFC analysis was conducted on MATLAB 2024a. For TMS stimulation target AF8, we defined a spherical ROI with a radius of 5 mm based on the Talairach coordinates (x = 43.9, y = 52.7, z = 9.3) reported by Koessler et al. (2009) to extract time series within right OFC^56^. For the rest of the brain, we extracted corresponding BOLD time series based on 264 functionally defined spherical ROIs (radius = 5 mm) in the Power atlas. For ROI discarding, we adopted 3 criteria: (1) ROIs with signal coverage less than 0.5 were excluded; (2) ROIs in the cerebellum region and undefined regions were excluded; (3) After discarding low-signal ROIs, ROIs within certain network with too few nodes (<10) and functionally unable to merge with other networks were excluded, which resulted in a number of 219 ROIs (one for OFC node) used for subsequent dFC matrix construction. We re-integrated the predefined functional networks from the Power atlas and finally examined the coupling of 8 functional networks with OFC targets.

For the retained ROIs, we extracted their time series and calculated the Pearson correlation between each pair of ROIs in a series of consecutive time windows. The length of each time window was set to 15 TR and moved to the next window with a step size of 1 TR. For each subject, all time windows were concatenated to form a dFC matrix of 219*219*156 (ROI*ROI*time window). Finally, we removed the negative connections in the dFC matrices and set all negative values to 0.

#### Community detection-based interregional integration

The community defines a connection pattern with stronger intra-connections than inter-connections. The multilayer community further assumes temporal continuity of the community architecture across windows, i.e., nodes are not only connected to nodes within the same window but also connected to the identical nodes in adjacent windows. In this study, the optimal modular architecture was identified by a multilayer-variant Louvain algorithm, which is defined as:

1 $${{{\boldsymbol{Q}}}}=\frac{1}{2\mu }{\sum }_{{ijlr}}\left[\left({A}_{{ijl}}-{\gamma }_{l}{V}_{{ijl}}\right){{{{\rm{\delta }}}}}_{{{{\rm{lr}}}}}+{\delta }_{{ij}}{\omega }_{{jlr}}\right]\delta ({g}_{{il}},{g}_{{jr}})$$

where *μ* denotes the total connection weight of the network, *A*_*ijl*_ denotes the connection between nodes *i* and *j* at layer *l* of the multilayer network, and *V*_*ijl*_ denotes the probability expected by chance of a connection corresponding *A*_*ijl*_. The variable *γ*_l_ sets the structural resolution parameter of layer *l*, e.g., the larger the value of *γ*_*l*_, the smaller the detected community size. The variable *ω*_*jlr*_ sets the temporal resolution parameter. Here we used a default setting: *γ*_*l*_ = 1, *ω*_*jlr*_ = 1. The variable *g* denotes the community assignments between node *i* in layer *l* and node *j* in layer *r*. The *δ* is a Kronecker delta function, where *δ(gil, gjr)* = 1 if *il* = *jr*; and 0 otherwise.

Given the heuristic nature of the multilayer modularity quality function *Q*^2^, we repeated 100 iterations for the multilayer community detection process. All community-relevant indicators were calculated as the average value across iterations.

After obtaining the community configuration of nodes in each time window, we calculated the module allegiance between right OFC nodes and 219 ROI nodes, generating a 1 × 219 matrix for each subject. Each element *P*_*ij*_ of the module allegiance matrix *P* denotes the relative frequency that subcortical node *i* and cortical node *j* were divided into the same community across windows. The element *P*_*ij*_ = 1 when nodes *i* and *j* are always in the same community and 0 when they are never in the same community. We then calculated the integration coefficient between the right OFC and the eight networks based on module allegiance. The integration of region *i* with respect to network *N*, ${I}_{i}^{N}$, is defined as:

2 $${I}_{i}^{N}=\frac{1}{{m}_{N}}{\sum }_{i\notin N,j\in N}{P}_{{ij}}$$

Where *m*_*Ns*_ denotes the number of ROI in network *N*. The variable ${I}_{i}^{N}$ denotes the average probability that the *i*^*th*^ region is in the same community as regions from the given network N. A region has a high integration with network N, indicating that it tends to communicate with N other than its own network across windows. The inter-network integration was obtained by averaging the ROI coefficient within the same network. *P*-value of group comparison was corrected by Bonferroni: p(corrected)=0.05/8 = 0.006.

#### Non-negative matrix factorization (NMF)

The NMF was used to parse the different patterns of interaction between OFC and ROIs within DMN. For all the subjects’ pre- and post-test data, the integration coefficients between OFC and 50 nodes within the DMN were used as inputs of NMF and concatenated to form a 168*50 non-negative matrix. NMF is favored for its ability to produce more interpretable, consistent, and precise results. Compared to traditional methods, NMF achieves a soft segmentation of pattern variation, which contributes to capturing complex brain states. This approach solves the matrix factorization problem (*V* ≈ *WH*, *W*&*H* ≥ 0) by optimizing a cost function:

3 $${\min }_{W,H}\frac{1}{2}{{{{\rm{||}}}}V-{WH}{{{\rm{||}}}}}_{F}^{2}$$

Where *V* is the 50*168 non-negative input matrix, *W* is a component matrix of OFC-DMN integration with size 50*k, and *H* is a weighted matrix of subject expression coefficient with size k*168. The parameter k is used to determine the number of components. We used a method called alternative non-negative least squares with block principal pivoting to solve this equation.

For the optimal parameter *k*, we used two criteria to determine: stability and residuals of *V*-*WH*. Specifically, for each *k* (from 2 to 15), NMF is run repeatedly with random initialization (100 times). If *k* is optimal, the resulting components should be stable across iterations. For each candidate value of *k*, we use the Hungarian matching algorithm to match the components across iterations and calculate the average Pearson correlation between the components. This process generates a 100 × 100 correlation matrix, where the elements represent the similarity of the components. We average this matrix to measure the stability of the k-based partitions. In addition, we calculated the residuals of *V*-*WH* in each iteration to characterize the difference between the decomposed matrix and the original matrix. Theoretically, if *k* is optimal, the corresponding stability value is the largest and the residual is the smallest, so the ratio of the stability to the residual is used to measure the optimal *k*. Finally, we selected the optimal parameter *k* = 10 and used it to average the results across 100 runs.

### Relationship between brain integration and genetics

We calculated the spatial associations of dynamic integration with the distribution of neuronal cell types obtained from human cell marker genes. To overcome recently reported non-negligible false positive rates when applying GCEA, we used ABAnnotate (https://github.com/LeonDLotter/ABAnnotate), a toolbox that applies a nonparametric method to estimate significant correlations relative to an ensemble of randomized phenotypes. ABAnnotate looks for genes with a positive correlation with a given phenotype, so we accounted for the polarity of the component map by applying the toolbox both to the original component map and the flipped component map. For each of the GCEA analyses, the toolbox first computes the enrichment score for each category by averaging across the Spearman rank correlation between each gene in a category and the component map. A null distribution of category enrichment scores is computed relative to the null component map, and a *p*-value is estimated by comparing the empirical category score to the null distribution and then false-discovery rate corrected (FDR < 0.05). In keeping with the previous analyses, we generated the randomized phenotypes by spinning a specific NMF component map 5,000 times.

### Inter-subject correlation

We first calculated the intervention differences (post-pre/post+pre) across participants on all sub-dimensions and concatenated them into a matrix. Specifically, the sub-dimensions at the neural level include the integration between OFC and DMN’s ROIs, forming a 45*50 matrix (subjects*ROI); the sub-dimensions at the symptom level include the 5 dimensions of PANSS, CGI, and GAF, forming a 45*10 matrix (because the symptom record includes 2 time points); the sub-dimensions at the cognitive level include the 8 dimensions of MCCB, ultimately forming a 45*8 matrix. For each matrix, we calculated the Pearson correlation across subjects along the sub-dimension direction and constructed a 45*45 ISC matrix. The elements in the ISC matrix represent the similarity between any pair of subjects on the sub-dimensions, and these elements are then mapped to the 0–1 interval. Finally, we calculated the correlation between the three ISC matrices to examine their synchronization.

### Granger causality

The bivariate Granger causality estimates the causal effect between two given time series via a linear autoregressive model. The Granger causality from time series *a* to *b* is defined as follows:

4 $${F}_{a\to b}={{\mathrm{ln}}}\frac{|\sum \left({\xi }_{t}\right)|}{{|\sum \left({\eta }_{t}\right)|}^{{{{\rm{T}}}}}}$$

Where residuals *ξ*_*t*_ and *η*_*t*_ correspond to the restricted and unrestricted regression models, respectively, and *∑* represents their variance. In this study, the time series between OFC-DMN integration was defined as a, and the time series between DMN and the other 7 networks was defined as b1 to b7, respectively. We examined the Granger causal effects of the OFC-DMN integration on 7 downstream time series.

### Data analysis

Group-level analysis used Mixed ANOVA to analyze the changes in clinical symptoms, cognitive function, and brain integration between the active and sham groups. Bonferroni correction was used to control false positives for the ANOVA results for both integration coefficients (*p* = 0.05/8) and NMF components (*p* = 0.05/10). Two-sample *t*-test and χ^2^ test were used to compare the age and sex differences between the two groups. Spearman correlation was used to calculate the correlation among clinical symptoms, cognitive function, and the brain network. Mediation and modulation analysis were used to test relationships among brain integration, cognitive function, and clinical symptoms. These statistical analyses were performed in R4.4.1, including lme4, bruceR, and ggwordcloud packages.

### Reporting summary

Further information on research design is available in the Nature Portfolio Reporting Summary linked to this article.

## Supplementary information

Supplementary Information

Peer Review file

Reporting Summary

## Source data

Source Data

## Data Availability

The dataset includes self-reported demographics, clinical assessments, neurocognitive measures, and functional neuroimaging data from individuals with first-episode psychosis. Although all identifying information has been removed, there remains a minimal risk of re-identification due to rare individual characteristics. To protect participant anonymity, the raw data are protected and are not available due to data privacy laws. The data are available upon request with a signed data-sharing agreement that ensures secure handling and storage in line with our protocol. Requests can be directed to the corresponding author (huqiang@sjtu.edu.cn) and will be addressed promptly. Access is limited to qualified researchers at recognized academic or medical institutions for non-commercial scientific purposes. Requests will normally be acknowledged within 2 weeks, with access granted within approximately 6–8 weeks. Besides, for all reported figures and table. Source data are provided with this paper.

## Code availability

Data post-processing was done in MATLAB. The dynamic community detection code is publicly available at https://github.com/GenLouvain/GenLouvain. The NMF code is publicly available at https://github.com/hiroyuki-kasai/NMFLibrary. The genotyping code is publicly available at https://github.com/LeonDLotter/ABAnnotate. Granger causality analysis used the dynamicBC toolbox. All statistical analyses were performed in R. Visualization was performed using BrainNet Viewer (https://www.nitrc.org/projects/bnv) and MRIcroGL (https://www.nitrc.org/projects/mricrogl/).
