# Supplementary material for: OFC-induced network modularity improves positive symptoms and attentional alertness in schizophrenia: a combined rTMS-fMRI study
Source: Nat Commun. 2026 May 30;17:7010. doi: 10.1038/s41467-026-72917-4 (PMC13392451; doi:10.1038/s41467-026-72917-4)
Supplement: Supplementary file 1 — Supplementary Information [file 41467_2026_72917_MOESM1_ESM.pdf]

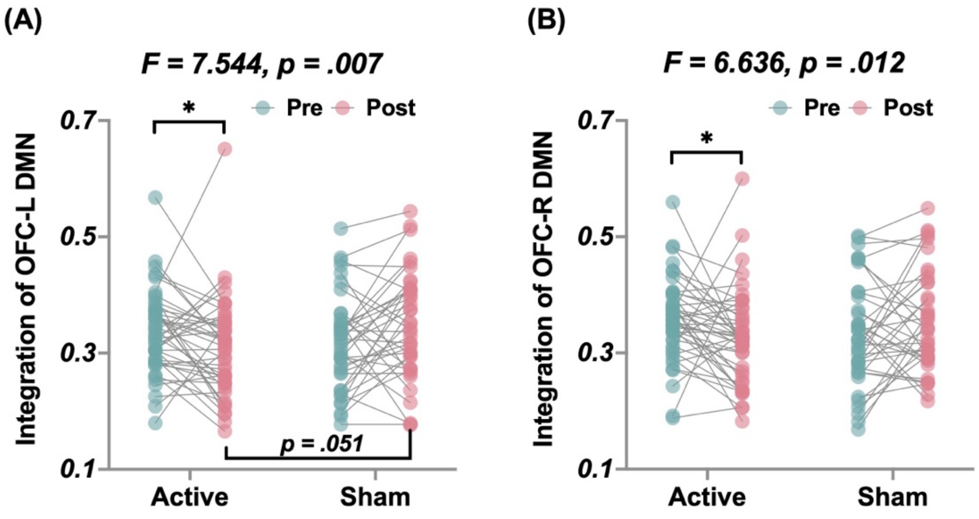

Fig. S1 Integration coefficients changes between OFC and the left DMN (A), and right DMN (B). Dots represent integration coefficients in the pre-intervention (green) and in the post-intervention (red). Gray lines connect data points from the same subject, illustrating individual changes between pre-intervention and post-intervention. Data are plotted as individual values; no error bars are shown. Sample size: n = 84 participants. Axes represent integration coefficient values (dimensionless). ANOVA analysis showed significant group differences in both hemispheres: left, $F(1,82) = 7.544$, $p = 0.007$, partial $\eta^2 = 0.08$, and right, $F(1,82) = 6.636$, $p = 0.012$, partial $\eta^2 = 0.07$, with reduced OFC–DMN integration in the active group but not in the sham group after rTMS treatment. Source data are provided as a Source Data file.

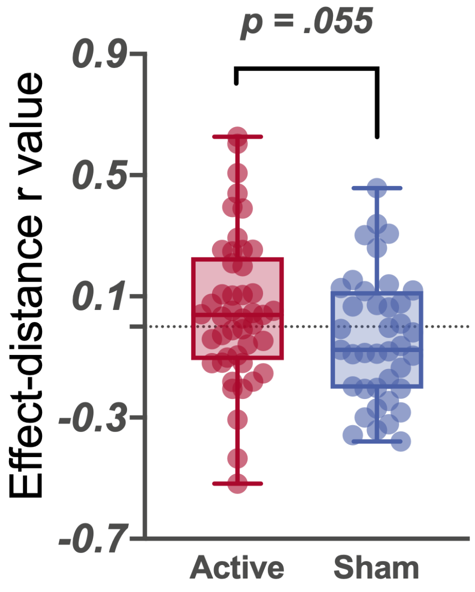

Fig. S2 Distance-dependent effects between the active and sham group at the individual level. Dots represent correlation r values between intervention effect and Euclidean distance in the active group (red) and sham group (blue). Sample size: n = 84. A two-sample t-test showed marginal significance (p=0.055). Exact p value is provided. No error bars are shown. Source data are provided as a Source Data file.

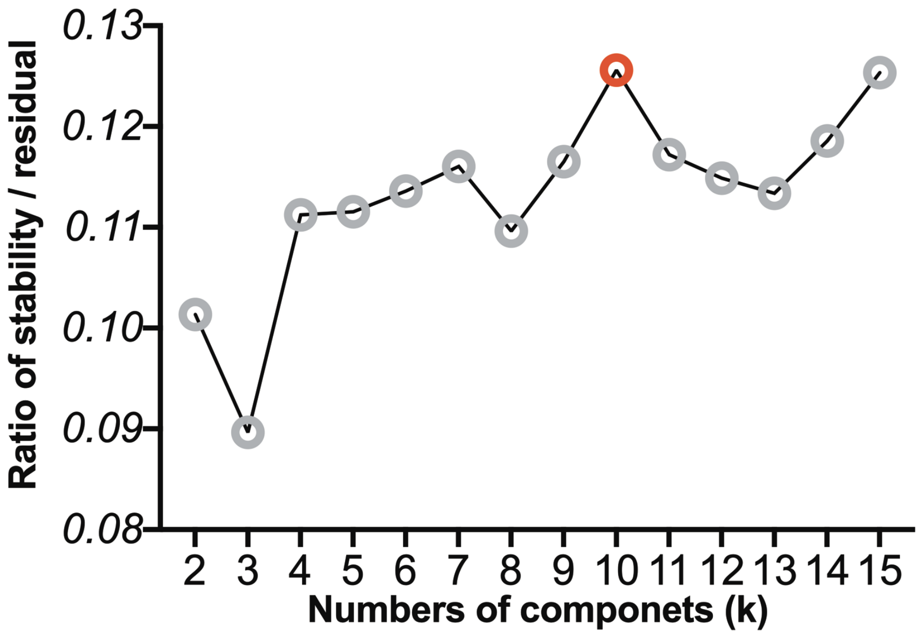

Fig. S3 Stability check of non-negative matrix factorization. Ten distinct components showed the best stability of non-negative matrix factorization. Source data are provided as a Source Data file.

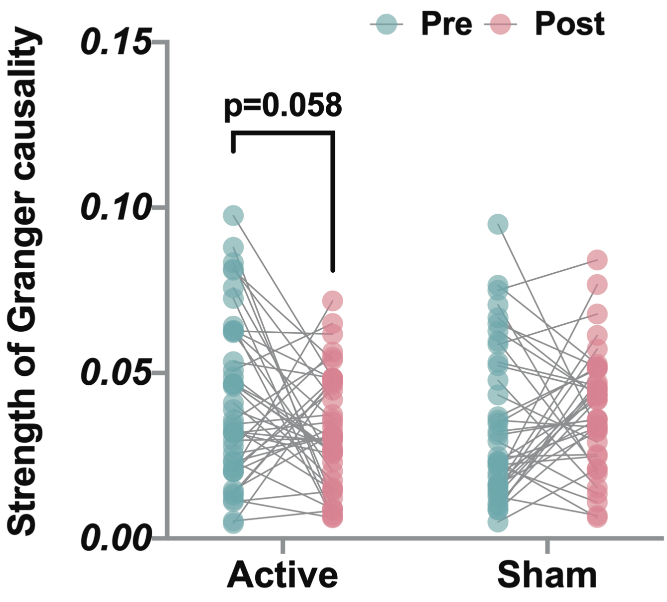

Fig. S4 The strength of Granger Causal influence from the OFC-DMN to the DMN-AN pathway. Dots represent individual participants. Values indicate Granger causal coefficients. This plot shows the simple effect after excluding two participants in the active group whose pretest causal influence exceeded 0.1. Final sample size: n=82. Exact p values are reported in the figure. Source data are provided as a Source Data file.

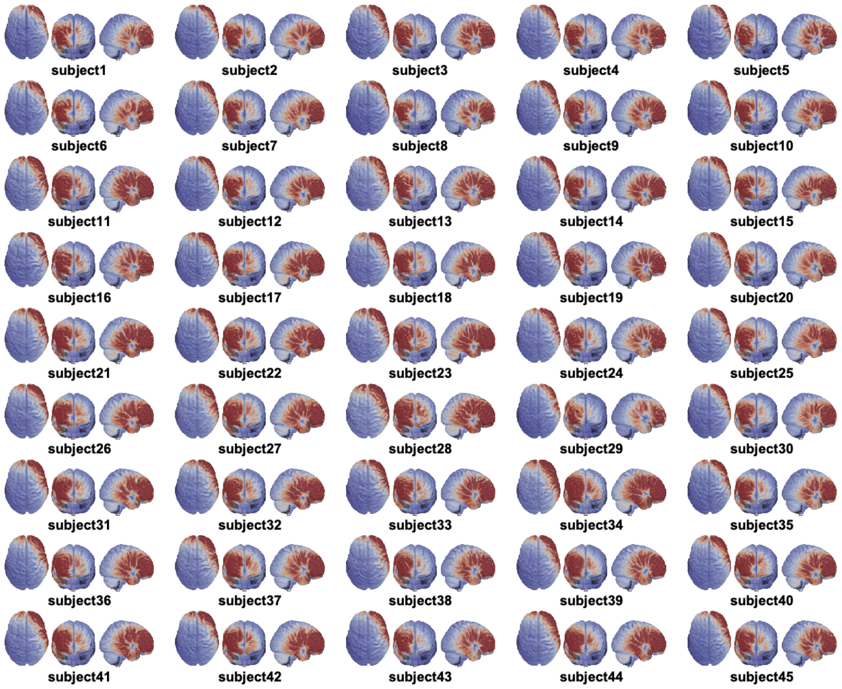

Fig. S5 The realistic calculations of the electric field through SimNIBS tools 43. Visualization shows simulated electric field magnitude (V/m). Color scale indicates electric field intensity values.
