# Supplementary material for: OFC-induced network modularity improves positive symptoms and attentional alertness in schizophrenia: a combined rTMS-fMRI study
Source: Nat Commun. 2026 May 30;17:7010. doi: 10.1038/s41467-026-72917-4 (PMC13392451; doi:10.1038/s41467-026-72917-4)
Supplement: Supplementary file 3 — Reporting Summary [file 41467_2026_72917_MOESM3_ESM.pdf]

nature portfolio

Corresponding author(s): Andre Aleman, Jijun Wang, Qiang Hu

Last updated by author(s): Oct 7, 2025

Reporting Summary

Nature Portfolio wishes to improve the reproducibility of the work that we publish. This form provides structure for consistency and transparency in reporting. For further information on Nature Portfolio policies, see our Editorial Policies and the Editorial Policy Checklist.

Statistics

For all statistical analyses, confirm that the following items are present in the figure legend, table legend, main text, or Methods section.

n/a	Confirmed	
☐	☒	The exact sample size (n) for each experimental group/condition, given as a discrete number and unit of measurement
☐	☒	A statement on whether measurements were taken from distinct samples or whether the same sample was measured repeatedly
☐	☒	The statistical test(s) used AND whether they are one- or two-sided<br>*Only common tests should be described solely by name; describe more complex techniques in the Methods section.*
☐	☒	A description of all covariates tested
☐	☒	A description of any assumptions or corrections, such as tests of normality and adjustment for multiple comparisons
☐	☒	A full description of the statistical parameters including central tendency (e.g. means) or other basic estimates (e.g. regression coefficient) AND variation (e.g. standard deviation) or associated estimates of uncertainty (e.g. confidence intervals)
☐	☒	For null hypothesis testing, the test statistic (e.g. F, t, r) with confidence intervals, effect sizes, degrees of freedom and P value noted<br>*Give P values as exact values whenever suitable.*
☒	☐	For Bayesian analysis, information on the choice of priors and Markov chain Monte Carlo settings
☒	☐	For hierarchical and complex designs, identification of the appropriate level for tests and full reporting of outcomes
☐	☒	Estimates of effect sizes (e.g. Cohen's d, Pearson's r), indicating how they were calculated

Our web collection on statistics for biologists contains articles on many of the points above.

Software and code

Policy information about availability of computer code

Data collection	Imaging data were acquired on a GE Discovery MR750 3Tesla MRI scanner system with 32-channel head coil. Clinical symptoms and cognitive function were recored with word paper.
Data analysis	Data preprocessing and denoising was performed by standard pipeline via fMRIPrep 23.1.4. Data post-processing was done in XCP-D and MATLAB(2024a). The dynamic community detection code is publicly available at https://github.com/GenLouvain/GenLouvain. The NMF code is publicly available at https://github.com/hiroyuki-kasai/NMFLibrary. The genotyping code is publicly available at https://github.com/LeonDLotter/ABAnnotate. Granger causality analysis used the dynamicBC toolbox. Genetics analysis was performed via ABAnnotate toolbox. All statistical analyses were performed in R4.4.1 including lme4, bruceR, ggwordcloud packages.

For manuscripts utilizing custom algorithms or software that are central to the research but not yet described in published literature, software must be made available to editors and reviewers. We strongly encourage code deposition in a community repository (e.g. GitHub). See the Nature Portfolio guidelines for submitting code & software for further information.

Data

Policy information about availability of data

All manuscripts must include a data availability statement. This statement should provide the following information, where applicable:

- Accession codes, unique identifiers, or web links for publicly available datasets
- A description of any restrictions on data availability
- For clinical datasets or third party data, please ensure that the statement adheres to our policy

The dataset includes self-reported demographics, clinical assessments, neurocognitive measures, and functional neuroimaging data from individuals with first-episode psychosis. Although all identifying information has been removed, there remains a minimal risk of re-identification due to rare individual characteristics. To protect participant anonymity, the data are available upon request with a signed data-sharing agreement that ensures secure handling and storage in line with our protocol. Requests can be directed to the corresponding author (huqiang@sjtu.edu.cn) and will be addressed promptly. Besides, for all reported figures and table, Source Data are provided with this paper.

Research involving human participants, their data, or biological material

Policy information about studies with human participants or human data. See also policy information about sex, gender (identity/presentation), and sexual orientation and race, ethnicity and racism.

Reporting on sex and gender	we aim to and are able to recruit equal numbers of males and females to control for biological sex of subjects. demographic information including sex are reported in Table1. Sex based analysis is not conducted in this study,
Reporting on race, ethnicity, or other socially relevant groupings	Ethnicity data was self-reported by subjects and was not analyzed in this study.
Population characteristics	We recruited a total of 98 subjects, with 53 in active group and 45 in sham group. in the formal analysis, in active group (include 45 subjects), 20 females and 25 males, mean age 27. in the sham group (39 subjects), 21 females and 18 males, mean age 27.
Recruitment	Participants were consecutively recruited from the inpatient and outpatient departments of the Harbin First Specialized Hospital between November 2019 and December 2021. Potentially eligible patients were screened by trained psychiatrists according to DSM-IV criteria. All eligible patients who met inclusion criteria during the recruitment period were invited to participate. The inclusion criteria included: (1) diagnosed with schizophrenia according to the DSM-IV diagnostic criteria, (2) willing to receive rTMS therapy and provide signed informed consent, (3) 15–45 years old and IQ>69, (4) drug-naïve and experiencing their first episode of psychosis, (5) positive and negative syndrome scale (PANSS) score ≥60, and (6) overall clinical global impression (CGI) scale ≥4. The subjects received atypical antipsychotics after enrollment. The exclusion criteria included: (1) sensorimotor disorders, neurological diseases, or other physical diseases, (2) rTMS treatment contraindications such as metal implants, obvious excitement, and irritability, (3) received ECT treatment within one month and (4) pregnancy. An independent third party sorted subjects into either the real or sham groups via computer-generated randomization. Potential sources of bias should be acknowledged. Recruitment from a single psychiatric hospital may limit the generalizability of the findings. In addition, participation required willingness to undergo rTMS treatment and provide informed consent, which may have led to underrepresentation of patients with poor insight, severe agitation, or lower treatment adherence. These factors should be considered when interpreting the applicability of the results to broader schizophrenia populations.
Ethics oversight	The study was approved by the Ethics Committee of Shanghai Mental Health Center(2017-24R1) and The First Psychiatric Hospital of Harbin(IRB2019-004)

Note that full information on the approval of the study protocol must also be provided in the manuscript.

Field-specific reporting

Please select the one below that is the best fit for your research. If you are not sure, read the appropriate sections before making your selection.

☒ Life sciences ☐ Behavioural & social sciences ☐ Ecological, evolutionary & environmental sciences

For a reference copy of the document with all sections, see nature.com/documents/nr-reporting-summary-flat.pdf

Life sciences study design

All studies must disclose on these points even when the disclosure is negative.

Sample size	Using G*POWER, set>95% power and alpha = 0.05 (two-tailed), effect size=0.2. the sample size of each group is 42. to make sure enough samples for analysis, we planed to recruit 50 subjects of each group.
Data exclusions	Fourteen subjects (8 subjects in the active group and 6 subjects in the sham group) could not complete the clinical assessment, cognitive assessment, or MRI scanning
Replication	we are seeking funding to continue conducting randomized clinical intervention using same intervention target and parameters.

Randomization	Subjects were randomly assigned to the active rTMS or sham rTMS group. An independent third party divided subjects into either the active or sham groups via computer-generated randomization.
Blinding	The clinical staff and subjects were blind to the assignment, except for one clinical technician who provided the rTMS or sham treatment according to the randomization numbers.

Reporting for specific materials, systems and methods

We require information from authors about some types of materials, experimental systems and methods used in many studies. Here, indicate whether each material, system or method listed is relevant to your study. If you are not sure if a list item applies to your research, read the appropriate section before selecting a response.

Materials & experimental systems

n/a	Involved in the study
☒	☐ Antibodies
☒	☐ Eukaryotic cell lines
☒	☐ Palaeontology and archaeology
☒	☐ Animals and other organisms
☐	☒ Clinical data
☒	☐ Dual use research of concern
☒	☐ Plants

Methods

n/a	Involved in the study
☒	☐ ChIP-seq
☒	☐ Flow cytometry
☐	☒ MRI-based neuroimaging

Clinical data

Policy information about clinical studies

All manuscripts should comply with the ICMJE guidelines for publication of clinical research and a completed CONSORT checklist must be included with all submissions.

Clinical trial registration	Chinese Clinical Trial Register Center (Registration number: ChiCTR2000041106)
Study protocol	Protocol will be shared when reasonable request
Data collection	The data were collected in the First Psychiatric Hospital of Harbin, China during 2019.2 to 2021.2
Outcomes	primary outcome: Positive and Negative Syndrome Scale; secondary outcome: cognitive function (MATRICS Test), MRI and EEG signals.

Plants

Seed stocks	*Report on the source of all seed stocks or other plant material used. If applicable, state the seed stock centre and catalogue number. If plant specimens were collected from the field, describe the collection location, date and sampling procedures.*
Novel plant genotypes	*Describe the methods by which all novel plant genotypes were produced. This includes those generated by transgenic approaches, gene editing, chemical/radiation-based mutagenesis and hybridization. For transgenic lines, describe the transformation method, the number of independent lines analyzed and the generation upon which experiments were performed. For gene-edited lines, describe the editor used, the endogenous sequence targeted for editing, the targeting guide RNA sequence (if applicable) and how the editor was applied.*
Authentication	*Describe any authentication procedures for each seed stock used or novel genotype generated. Describe any experiments used to assess the effect of a mutation and, where applicable, how potential secondary effects (e.g. second site T-DNA insertions, mosiacism, off-target gene editing) were examined.*

Magnetic resonance imaging

Experimental design

Design type	resting-state functional MRI
Design specifications	not related
Behavioral performance measures	not related

Acquisition

Imaging type(s): functional and structual

Field strength: 3T

Sequence & imaging parameters: Anatomical images for registration to Montreal Neurological Institute (MNI) template space were acquired for each participant using a T1-weighted Magnetization acquisition with Spin Echo sequence: TR=8.20ms, TE=3.22ms, voxel size=1×1×1mm, flip angle=12°, field of view (FOV)=256mm. Functional images were acquired with using gradient-echo echo-planar imaging (EPI) with following parameter: TR=2000ms, TE=45ms, FOV=200mm, thickness=4mm, slices=32, flip angle=90°, voxel size 2 ×2 ×2mm

Area of acquisition: whole brain

Diffusion MRI: ☐ Used ☒ Not used

Preprocessing

Preprocessing software: Both structural and functional MRI data were minimally preprocessed using fMRIPrep 23.1.4 based on Nipype 1.8.6.

Normalization: Anatomical data preprocessing: Spatial normalization was performed using nonlinear registration with the ANTs antsRegistration tool, aligning the brain-extracted T1w-reference to the MNI space (MNI152NLin6Asym).

Normalization template: MNI space (MNI152NLin6Asym).

Noise and artifact removal: To account for confounding factors, framewise displacement (FD), DVARS, and three region-wise global signals were calculated. Both FD and DVARS were calculated for each functional run using their implementations in Nipype. The three global signals were extracted from the cerebrospinal fluid (CSF), white matter (WM), and whole-brain masks. Additionally, physiological regressors were extracted to enable component-based noise correction (CompCor), including temporal (tCompCor) and anatomical (aCompCor) variants.

Volume censoring: Volumes with filtered FD greater than 0.5 mm were cubic spline interpolated in the BOLD data.

Statistical modeling & inference

Model type and settings: Dynamic functional connectivity,Community detection-based interregional integration and Non-negative matrix factorization were calculated in the first level and then performed ANOVA group analysis

Effect(s) tested: ANOVA analysis

Specify type of analysis: ☐ Whole brain ☒ ROI-based ☐ Both

Anatomical location(s): based on 264 functionally defined spherical ROIs (radius = 5 mm) in the Power atlas.

Statistic type for inference (See Eklund et al. 2016): cluster-wised inference

Correction: Bonferroni correction was used to control false positives for the ANOVA results for both integration coefficients (p = 0.05/8) and NMF components (p = 0.05/10).

Models & analysis

n/a	Involved in the study
☐	☒ Functional and/or effective connectivity
☒	☐ Graph analysis
☒	☐ Multivariate modeling or predictive analysis

Functional and/or effective connectivity: pearson correlation
