# Supplementary material for: OFC-induced network modularity improves positive symptoms and attentional alertness in schizophrenia: a combined rTMS-fMRI study
Source: Nat Commun. 2026 May 30;17:7010. doi: 10.1038/s41467-026-72917-4 (PMC13392451; doi:10.1038/s41467-026-72917-4)
Supplement: Supplementary file 2 — Peer Review file [file 41467_2026_72917_MOESM2_ESM.pdf]

nature portfolio

Peer Review File

OFC-induced network modularity improves positive symptoms and attentional alertness in schizophrenia: a combined rTMS-fMRI Study

Corresponding Author: Ms Ningning Zeng

This file contains all reviewer reports in order by version, followed by all author rebuttals in order by version.

Version 0:

Reviewer comments:

Reviewer #1

(Remarks to the Author)
The authors present an RCT of a 20-session course of active vs sham TMS to the right orbitofrontal cortex (OFC) in schizophrenia, demonstrating improvements in negative and cognitive symptoms in particular, as well as a lesser improvement in positive symptoms. fMRI analyses suggested that active stimulation reduced network 'integration' between the default mode network and the right OFC, which is in keeping with previous work suggesting that the right OFC serves as a nexus of 'non-reward' function within the brain as a whole.

This ambitious study contains several important innovative features including: 1) the use of a relatively under-explored but potentially very fruitful TMS target in the right OFC; 2) the application of therapeutic TMS not in common indications such as depression but rather in schizophrenia, an under-studied but potentially very impactful TMS indication; 3) the use of graph-theoretical measures applied to resting-state fMRI in order to understand the neural correlates of symptom improvement, and 4) the incorporation of genetic factors into the analysis of the neuroimaging data. Each of these features bolsters the potential interest and impact of the study. The use of an RCT design, a relatively adequate 20-session TMS course, and a relatively large ITT sample size of 98 patients also place this study well ahead of many previous efforts in the TMS literature on this population. Finally, the demonstration of a significant effect on negative symptoms is very encouraging, as conventional treatments are generally quite poor in treating these symptoms.

The manuscript is for the most part clearly written; the background and discussion are appropriately referenced, and the findings are potentially of great interest to the field. I do have a few minor suggestions for revisions:

- Figure 5B: these timeseries of integration coefficients are reported to represent group data, although resting-state fMRI timeseries are more usually associated with individual subjects rather than groups. Further explanation for the general reader (either in the legend or the main text) may be useful to explain the appropriateness of this type of plot for group results rather than individual results.

- Figure 5C: the distributions of the individual subjects' Granger causality values, as shown here, suggest that the active group and the sham group actually had quite a difference at baseline, before treatment. Indeed, all of the values for 3 out of the 4 measurement points appear under 0.10, and only the active group at baseline has any values above 0.10. In this context, the reduction of Granger causality values in the active but not the sham group could potentially reflect floor effects or regression to mean, rather than treatment effect. Since this analysis is acknowledged to be exploratory in nature, such ambiguities are not fatal to the core findings of the study, but they should be acknowledged at he very least.

- Discussion: "Encouraged by this, we further focused on specific intervention pathways and found that alterations in brain integration selectively improved attention cognition with moderated by positive symptom." - this sentence is not grammatical and needs correcting.

- Discussion: "the medial prefrontal cortex, an area of the DMN in participants at ultra-high risk for psychosis." - this sentence does not make sense as written, and I suspect a comma is needed after "DMN"

- Discussion: "Excessive integration between OFC and DMN may blur the normal boundary between internal thoughts and external environments, lead to an exaggerated focus on one's own thoughts and feelings." This is a possible interpretation, but it leaves out the important role of the right OFC in non-reward functions (e.g., Rolls, 2016; PMID: 27181908). For patients in whom negative symptoms are improving, an important mechanism may be the uncoupling of excessive non-reward activity from the activity of the DMN. A non-reward-based interpretation of the findings on OFC connectivity and negative symptoms should be included in the Discussion as another possibility.

- Methods: "The clinical staff and patients were blind to the assignment, except for one clinical technician who provided the rTMS or sham treatment according to the randomization numbers." - this sentence appears in two sections, and should be eliminated from "Participants"

- Methods: "with the coil vertically positioned over the AF8 site": more clarity is needed: does this mean that the handle was downwards, so that the coil current was directed upwards, at right angles to the line of the head circumference, as in Feffer et al. or Fettes et al among the references? The exact orientation and direction of the coil and current should be indicated.

- Methods: were there any scalp electrodes placed near the coil to mimic the scalp sensations in the sham group? These electrodes are controversial and not necessarily required, but it should be made explicit as to whether they were present or not. Was a MagVenture sham coil used (if so, what model number) or was it a custom-built apparatus?

Reviewer #2

(Remarks to the Author)
This manuscript was generally well-written. It's a well-designed RCT to explore the efficacy and neural mechanisms of 1Hz rTMS over the right OFC in first-episode, drug-naïve people with schizophrenia. It combined with rs-fMRI and proposed a multi-level causal pathway analysis. It's a timely contribution to the current topics of precision medicine in psychiatry. I have several comments/suggestions for further possible improvement of the manuscript.

1.After demonstrating the DMN differences, the authors distinguished between the anterior and posterior parts of the DMN for analysis. Given the location of rTMS stimulation, why is it not consider to compare between left and right sides? It may be clinically reasonable.
2.In the previous rTMS studies, high frequency parameters (eg. 5Hz rTMS targeting for left DLPFC) were often employed. It's especially reported for the improvement of emotional symptoms. In the present trial (a novel combination of parameter and targeting area), the two arms were 1Hz rTMS and sham control over the right OFC, is there any emotional symptom related variable recorded?
3.Please provide the trial registration number;
4.It needs detailed evidence based description or critical comment, eg. In the second paragraph of background part, TMS has played 'an invaluable role';
5.In the viewpoint of public mental health, I'd like to recommend use people with schizophrenia instead of 'patient with schizophrenia' in the manuscript.

Reviewer #3

(Remarks to the Author)

Zeng et al investigate rTMS-induced effects on OFC connectivity and its relationship to symptomatic and cognitive improvements in patients with schizophrenia. The authors conduct a range of interesting analyses and offer a comprehensive model of the mechanistic pathway for the effectiveness of OFC stimulation in the disorder.
A few points of clarification on the methods are warranted; aside from this I have mostly minor comments.

Introduction
- The introduction is generally well written, but would benefit from some additional proof-reading to improve on some somewhat vague phrases in several places (e.g. "noninvasive transcranial brain technique"; "causal structure of brain and behavioral symptoms")

- It would be interesting to understand a little more about what is known of the clinical efficacy and network dynamics of OFC stimulation outside of the current study. Could the authors briefly expand on any findings in other datasets investigating the effects of OFC rTMS, if available?

Methods and results
- Could the authors expand on why negative correlations in the dFC matrices were set to 0?

- Could the authors expand on how corrections for multiple comparisons were applied? The methods state that "P values of group comparison was corrected by Bonferroni" – is this referring to the fact that group x session interactions are investigated for connectivity between OFC and 8 different networks? Further,10 NNMF components are tested for the interaction of interest – are corrections applied at this level, too? Similarly, the "Data analysis" section later simply states that "Multiple comparison used Bonferroni correction", which is insufficient information as it is unclear what (how many models)

this refers to.

- It is not entirely clear what motivated the specific focus on the attention network. Presumably this is based on findings related to attention/vigilance (AV) scores. The authors appear to be using “cognitive alertness” as a conceptual synonym for AV – this will need to be explicitly mentioned in order to make the link to the attention network analyses. In addition, the statement “Cognitive alertness is a common manifestation of positive symptoms and tends to be more connected to the attention network” needs refining – it is unclear in how far cognitive alertness is a “manifestation” of positive symptoms, and please provide a reference to its (intuitive) link to the attention network.

- The statistical language is at time imprecise, e.g. “interaction of integration between the right OFC and DMN between group and session was significant” – do the authors mean “the interaction between group and session on integration between right OFC and DMN was significant”?

Minor comments:
- “with matched the age and sex” occurs several times in the manuscript, please correct
- The input to NMF is described as the “difference of integration coefficients” – please be explicit that this refers to the difference between pre and post treatment
- Fig 4 legend typo: “mediaction”

Reviewer #4

(Remarks to the Author)
This study aimed to provide a more comprehensive understanding of how brain stimulation functions under repetitive transcranial magnetic stimulation (rTMS) treatment. The authors seek to transform rTMS from a “black-box” intervention into a mappable, observable therapeutic process, supported by direct visual evidence of network-level effects.
The exceptional novelty lies in the mechanistic transparency achieved through dynamic community detection methods. Researchers and clinicians observed how brain networks reorganize during treatment, watching therapeutic effects propagate both spatially and temporally from the stimulation site through connected brain systems. The dynamic integration coefficient visualizations provide insights into therapeutic network reconfiguration, showing how orbitofrontal cortex (OFC) stimulation reduces pathological integration with the default mode network (DMN) over time. This level of mechanistic visualization has never been demonstrated in previous rTMS-OFC studies and represents a paradigm shift toward mechanism-guided therapy.
The visual biomarkers showed here might enable treatment monitoring, allowing clinicians to track patient responses by observing network integration patterns during treatment. The distance-dependent effect provide a spatial map of treatment influence, potentially guiding optimal coil placement and stimulation parameters. The integration of genetic expression data with treatment-responsive brain regions offers a novel framework for personalized medicine, where baseline patterns might predict treatment response.
The choice of orbitofrontal cortex as a stimulation target represents a significant innovation, departing from conventional dorsolateral prefrontal and temporoparietal approaches. The authors demonstrate that OFC stimulation produces spatially constrained effects that cascade through specific network pathways, ultimately improving both positive symptoms and cognitive function. The comprehensive analytical framework spanning genes, networks, cognition, and symptoms shows how modern neuroscience methods can be integrated to understand complex treatment mechanisms at multiple biological levels. Their demonstration that reduced OFC-DMN integration corresponds with clinical improvements offers a concrete therapeutic target for future treatment development. The temporal dynamics suggest that network-based biomarkers could optimize stimulation protocols and predict individual outcomes.
The fundamental contribution of making rTMS mechanisms visible and quantifiable represents a transformative advance establishing new standards for understanding neuromodulation effects. However, important technical limitations warrant consideration in future clinical applications.
Major Concerns
1) The central premise of this study, reduced OFC-DMN integration represents beneficial "modularity", requires more substantial theoretical and empirical justification. Throughout the manuscript, the authors consistently interpret decreased integration as therapeutically advantageous, assuming a direct causal pathway from reduced integration to improved modularity to better clinical outcomes. However, this interpretation rests on several unexamined assumptions that weaken the overall argument. The paper would benefit from addressing why reduced integration should be considered inherently beneficial rather than potentially reflecting network disruption or dysfunction. Without healthy control comparisons to establish baseline integration levels, it remains unclear whether the observed OFC-DMN integration in patients is indeed pathologically excessive or within normal ranges. The authors should consider that optimal brain function may require balanced rather than minimal integration, and that the therapeutic effects could result from normalization rather than simple reduction of connectivity.
2) The study presents a significant logical inconsistency between its mediation and moderation analyses that undermines the coherence of the proposed mechanism. The mediation analysis suggests that cognitive improvements mediate the relationship between brain connectivity changes and symptom improvement, positioning cognition as an intermediate step in a linear causal chain. However, the moderation analysis indicates that positive symptoms moderate the relationship between brain integration changes and attention improvements, suggesting an interactive rather than mediating role for symptoms. These findings represent fundamentally different causal models that cannot both be correct simultaneously. The authors acknowledge this discrepancy in lines 411-417 but fail to provide a satisfactory resolution or explanation. This inconsistency raises questions about the stability and interpretability of the observed relationships and suggests the need for more careful theoretical modeling of how brain changes, cognitive improvements, and symptom reductions interact following

rTMS intervention.
3) The genetic association analysis, while intriguing, suffers from post-hoc reasoning that limits its mechanistic interpretability. The authors correlate gene expression maps with treatment-induced changes in brain integration patterns and conclude that the observed effects are "supported by underlying excitatory neurotransmission and expression of schizophrenia-related genes." However, this approach demonstrates correlation rather than causation and doesn't establish that genetic factors predict treatment response or drive the therapeutic mechanisms. The temporal logic is problematic: genetic expression patterns are used to explain treatment-induced changes, but no evidence is provided that these genetic factors influenced treatment response prospectively. The analysis would be more appropriately framed as exploratory validation of the biological plausibility of the findings rather than mechanistic evidence for the treatment effects.
4) The Granger causality analysis presents additional methodological concerns that may compromise the validity of the mechanistic interpretations. The authors apply Granger causality to integration coefficients derived from dynamic network measures rather than to raw time series data, which may not reflect true temporal causality relationships. Furthermore, the interpretation that reduced causal influence from OFC-DMN to DMN-Attention networks is beneficial could alternatively indicate breakdown of important regulatory mechanisms. The limited temporal resolution (156 time windows from 4-minute scans) may be insufficient for robust causality inference, particularly for the complex, derived measures used in this analysis. The authors should provide stronger justification for this analytical approach and consider alternative interpretations of reduced causality that don't assume therapeutic benefit.
5) Several aspects of the statistical analysis raise concerns about the reliability and generalizability of the findings. The study examines multiple brain networks (8 networks), numerous cognitive domains, and various symptom subscales, creating substantial multiple comparison issues that are only partially addressed through Bonferroni correction for network-level analyses. The complex moderation analyses are conducted on a relatively small active treatment group (n=45), which may be underpowered to detect reliable interaction effects, particularly given the number of variables examined. The authors report p-values extensively but provide limited information about effect sizes, making it difficult to assess the clinical significance of the observed changes. Additionally, the 4-week intervention period with immediate post-treatment assessment provides no information about the stability or durability of the observed effects, limiting conclusions about therapeutic utility.
6) The paper lacks evidence for the specificity of the observed effects to OFC stimulation, which weakens claims about the unique therapeutic potential of this target. Without comparison to other stimulation sites, it remains unclear whether the observed network changes represent OFC-specific mechanisms or general effects of prefrontal stimulation. The distance-dependent effects described in the results are presented as supporting evidence for OFC-specific influence, but the underlying mechanism for why proximity should determine integration changes is not adequately explained. The authors should acknowledge this limitation or provide stronger theoretical and empirical support for OFC-specific mechanisms.
7) The theoretical framework connecting reduced OFC-DMN integration to improved attention and reduced positive symptoms requires more rigorous development. The paper assumes that DMN hyperactivation interferes with attention networks and that reducing this interference is beneficial, but this simplified model may not capture the complexity of real brain network interactions. Networks are not purely competitive systems, and reducing communication between them could impair rather than improve function. The authors should consider alternative models where optimal function requires balanced rather than minimal inter-network communication and address how their findings fit within broader theories of network dysfunction in schizophrenia.
8) The authors should provide more conservative interpretations of their correlation-based findings, particularly regarding genetic associations and network causality. The study would benefit from acknowledging that observed associations establish relationships rather than mechanisms and that the proposed causal chains require additional validation. Future studies could strengthen these claims by including healthy control groups for baseline comparisons, longer follow-up periods to assess stability, and direct comparisons with other stimulation targets to establish specificity.
9) The figure legends throughout the manuscript lack sufficient detail for independent interpretation and fail to meet the standards expected for a high-impact journal. Many legends assume readers are familiar with the specific methodologies and abbreviations used, making the figures difficult to understand without repeatedly referencing the methods section. The legends should be comprehensive enough that readers can understand the main findings from the figures alone, following the principle that figures should be self-contained units of scientific communication. The figure legends consistently fail to provide fundamental information necessary for independent interpretation. Technical terms and abbreviations are used without definition, leaving readers to guess at meanings or search through the methods section for clarification. Color schemes, symbols, and scaling information are routinely omitted, making it impossible to interpret the visual data being presented. Sample sizes are rarely specified for individual analyses, and when statistical significance indicators are used, their meanings and thresholds are not explained. Units of measurement are frequently absent from quantitative displays, and perhaps most importantly, the legends provide no biological or clinical interpretation of what significant findings actually mean in the context of the research question. This lack of comprehensive detail forces readers to constantly cross-reference between figures, methods, and results sections, significantly reducing the accessibility and impact of the visual presentations. The legends should serve as complete, self-contained explanations that allow readers to understand both the technical aspects of the analysis and the scientific significance of the findings without requiring extensive background reading.
For example: Figure 2: The current legend simply states "group × session interaction of integration in 8 networks" without explaining what "integration" means in this context, how it was calculated, or what the color coding represents. The legend should specify that integration refers to "the average probability that the OFC is in the same community as nodes from each network across time windows" and explain that higher values indicate greater cross-network communication. The violin plots in panels B-D lack explanation of what the individual points represent (are these individual subjects or time windows?), and the color scheme is not defined. The correlation plots in panels E-G need clearer explanation of what "Euclidean distance" refers to (maybe physical distance in mm between brain regions? This even did not mention in Methods) and why this relationship is theoretically important. I cannot find the relevant descriptions in Results or Discussions.

Version 1:

Reviewer comments:

Reviewer #1

(Remarks to the Author)
The authors have made comprehensive and useful revisions to the manuscript following reviewer feedback.

I do not have any further revisions to suggest.

Reviewer #2

(Remarks to the Author)
The authors replied all the questions and comments, and revised it accordingly. There were two suggestions for minor revision:
1.In the abstract, the conclusion should revised more conservative interpretations accordingly. “the critical role” - (“These findings reveal the critical role for the dynamic modularity of the OFC as an “intermediate phenotype” mediating the pathway from genetic variation to behavioral manifestations, ”)
2.In the background part: In the second paragraph of background part, TMS has played ‘an invaluable role’, from the related evidence, TMS for schizophrenia doesn’t reach ‘an invaluable role’, it’s better to use other proper word/expression.

Reviewer #3

(Remarks to the Author)
The authors have satisfactorily addressed my concerns, and I am happy to recommend this for publication.

Open Access This Peer Review File is licensed under a Creative Commons Attribution 4.0 International License, which permits use, sharing, adaptation, distribution and reproduction in any medium or format, as long as you give appropriate credit to the original author(s) and the source, provide a link to the Creative Commons license, and indicate if changes were made.
In cases where reviewers are anonymous, credit should be given to 'Anonymous Referee' and the source.
The images or other third party material in this Peer Review File are included in the article’s Creative Commons license, unless indicated otherwise in a credit line to the material. If material is not included in the article’s Creative Commons license and your intended use is not permitted by statutory regulation or exceeds the permitted use, you will need to obtain permission directly from the copyright holder.
To view a copy of this license, visit https://creativecommons.org/licenses/by/4.0/

Reviewer #1:

The authors present an RCT of a 20-session course of active vs sham TMS to the right orbitofrontal cortex (OFC) in schizophrenia, demonstrating improvements in negative and cognitive symptoms in particular, as well as a lesser improvement in positive symptoms. fMRI analyses suggested that active stimulation reduced network 'integration' between the default mode network and the right OFC, which is in keeping with previous work suggesting that the right OFC serves as a nexus of 'non-reward' function within the brain as a whole.

This ambitious study contains several important innovative features including: 1) the use of a relatively under-explored but potentially very fruitful TMS target in the right OFC; 2) the application of therapeutic TMS not in common indications such as depression but rather in schizophrenia, an under-studied but potentially very impactful TMS indication; 3) the use of graph-theoretical measures applied to resting-state fMRI in order to understand the neural correlates of symptom improvement, and 4) the incorporation of genetic factors into the analysis of the neuroimaging data. Each of these features bolsters the potential interest and impact of the study. The use of an RCT design, a relatively adequate 20-session TMS course, and a relatively large ITT sample size of 98 patients also place this study well ahead of many previous efforts in the TMS literature on this population. Finally, the demonstration of a significant effect on negative symptoms is very encouraging, as conventional treatments are generally quite poor in treating these symptoms.

The manuscript is for the most part clearly written; the background and discussion are appropriately referenced, and the findings are potentially of great interest to the field. I do have a few minor suggestions for revisions:

- Figure 5B: these timeseries of integration coefficients are reported to represent group data, although resting-state fMRI timeseries are more usually associated with individual subjects rather than groups. Further explanation for the general reader (either in the legend or the main text) may be useful to explain the appropriateness of this type of plot for group results rather than individual results.

Response: We thank the reviewer for this helpful suggestion. As noted, Fig. 5B shows the time series of the group-level integration coefficients, which are intended for illustrative purposes. In the actual Granger causality analysis, we used the individual-level time series. We have now clarified this point in both the main text and the figure legend to avoid potential misunderstanding.

1）Results section [Page 13, Line 8-10]

"Mixed-design ANOVA analysis showed a significant group × session interaction in causality across OFC-DMN-AN (F = 6.307, p = 0.014, *partial* η^2=0.07; Fig. 5A). Fig. 5B shows the group-level time series before and after the intervention."

2）Figure legend of Fig.5B [Page 14, Line 7-9]

"(B) Group-average time series of integration coefficients for the OFC-DMN (red) and DMN-AN (blue) pathways, before and after the intervention for both active (top) and sham (bottom) groups. Shaded areas represent the standard error of the mean."

- Figure 5C: the distributions of the individual subjects' Granger causality values, as shown here, suggest that the active group and the sham group actually had quite a difference at baseline, before treatment. Indeed, all of the values for 3 out of the 4 measurement points appear under 0.10, and only the active group at baseline has any values above 0.10. In this context, the reduction of Granger causality values in the active but not the sham group could potentially reflect floor effects or regression to mean, rather than treatment effect. Since this analysis is acknowledged to be exploratory in nature, such ambiguities are not fatal to the core findings of the study, but they should be acknowledged at he very least.

Response: We appreciate the reviewer for pointing out details that we overlooked. The data point distribution in Fig.5C seems to have extreme values, which reduces the Granger causality. Therefore, we attempted additional analysis to clarify this issue.

First, we calculated the mean and standard deviation (0.0457±0.030) of the active group's pre-test data to confirm whether there were any extreme values (> ±3*SD). The results showed that one participant's value (0.143) exceeded the threshold of 3*SD, indicating the presence of an extreme value (Pukelsheim, 1994).

Second, we deleted the data (2 cases) with a pre-test score exceeding 0.1 in the active group and used the remaining 82 subjects for repeated measures ANOVA. The results showed a significant Group × Session interaction for causal relationships in the OFC-DMN-AN (F = 4.352, p = 0.040). Post hoc analysis showed that the reduction in Granger causality in the active group was marginally significant (t = 1.950, p = 0.058).

Combined with these results, we believe that extreme values in the active group do lead to a more significant reduction in Granger causality, but after removing extreme values, the interaction effect is still significant.

To better clarify this issue in the manuscript, we point out the extreme value problem and conduct further robustness analysis and add a supplementary figure in the Results section as below:

Results section [Page 13, Line 15-26]

"In addition, we observed that the reduced causal influence may be affected by the extreme values of the active group in the baseline. We therefore excluded the two subjects in the active group whose pretest causal influence exceeded 0.1 and repeated the analysis with the remaining subjects. The results still showed a significant interaction (F = 4.352, p = 0.040, *partial* η^2=0.05), and the reduction of Granger causality in the active group was close to significant (t = 1.950, p = 0.058, *Cohen's d* = 0.29, see supplementary Fig.S4). After accounting for potential regression to the mean, our analysis provides preliminary evidence suggesting that the rTMS-induced modularity between the OFC and DMN may attenuate the DMN's causal influence on the downstream AN. Although this attenuation is consistent with the inhibitory effects of 1-Hz rTMS and our overall hypothesis, we should interpret this finding with caution: this could also reflect the disruption of a potentially important regulatory mechanism, a possibility that our data cannot exclude."

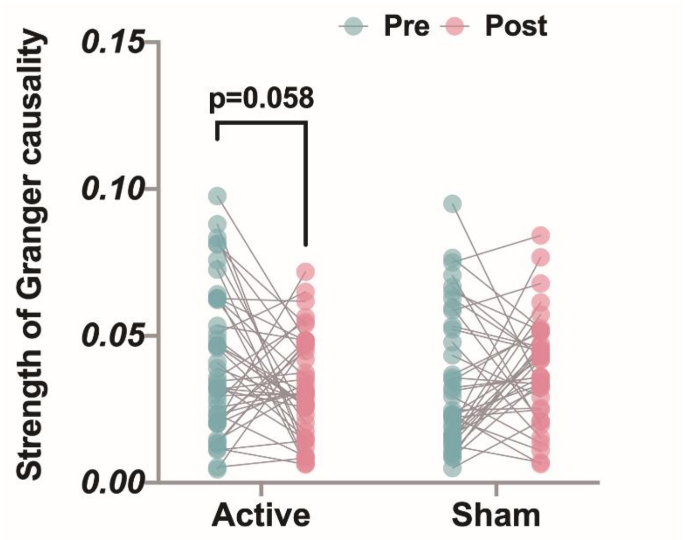

Fig. S4 The strength of Granger Causal influence from the OFC-DMN to the DMN-AN pathway. This plot shows the simple effect after excluding two participants in the active group whose pretest causal influence exceeded 0.1.

Reference:

Pukelsheim, Friedrich. "The three sigma rule." The American Statistician 48.2 (1994): 88-91.

- Discussion: "Encouraged by this, we further focused on specific intervention pathways and found that alterations in brain integration selectively improved attention cognition with moderated by positive symptom." - this sentence is not grammatical and needs correcting.

Response: We thank the reviewer for pointing out this grammatical error. We have corrected the sentence in the revised manuscript. The revised sentence now reads:

Discussion section [Page 15, Line 9-11]

"Encouraged by this, we further focused on specific intervention pathways and found that alterations in brain integration selectively improved attentional performance, with this improvement being moderated by positive symptoms."

- Discussion: "the medial prefrontal cortex, an area of the DMN in participants at ultra-high risk for psychosis." - this sentence does not make sense as written, and I suspect a comma is needed after "DMN"

Response: We thank the reviewer for pointing out this grammatical error. We have revised the sentence in the manuscript to include the comma, and it now reads:

Discussion section [Page 15, Line 28-30]

"They report increased connectivity between the SN and the medial prefrontal cortex, an area of the DMN, in participants at ultra-high risk for psychosis."

- Discussion: "Excessive integration between OFC and DMN may blur the normal boundary between internal thoughts and external environments, lead to an exaggerated focus on one's own thoughts and feelings." This is a possible interpretation, but it leaves out the important role of the right OFC in non-reward functions (e.g., Rolls, 2016; PMID: 27181908). For patients in whom negative symptoms are improving, an important mechanism may be the uncoupling of excessive non-reward activity from the activity of the DMN. A non-reward-based interpretation of the findings on OFC connectivity and negative symptoms should be included in the Discussion as another possibility.

Response: We sincerely thank the reviewer for this insightful comment. The non-reward function of the orbitofrontal cortex (OFC) is indeed a plausible mechanistic explanation. In our initial discussion, because we only observed a three-way correlation among positive symptoms, attentional vigilance, and OFC-DMN integration, we focused on this specific change as the primary explanatory outcome. However, from a symptomatic outcome alone, the OFC-directed physiological effects also reduced negative symptoms as well. For patients in whom negative symptoms are improving, a decoupling of excessive non-rewarding activity from DMN activity is indeed an explanation that needs to be considered.

We have revised the Discussion section to include this important possibility. We now propose that the observed excessive integration between the OFC and the DMN, particularly in the context of negative symptoms, could also reflect an over-representation of non-reward signals (e.g., punishment, non-delivery of rewards) within the DMN. This could lead to a persistent,

negative, and self-focused emotional bias, thereby contributing to the experience of negative symptoms. In this framework, the observed improvement in negative symptoms following the intervention could thus be interpreted as the uncoupling of this pathological non-reward activity from the DMN.

We have now added a new paragraph in the Discussion to reflect this interpretation, citing the suggested reference (Rolls, 2016, PMID: 27181908) as below:

Discussion section [Page 15, Line 40-43 to Page 16, Line 1-8]

"An alternative, yet complementary, interpretation of our findings on OFC-DMN connectivity relates to the well-established role of the right OFC in processing non-reward signals[43]. This theory posits that the OFC contains specialized neurons that, in response to non-reward or punishment, can enter a persistent "attractor state" maintaining their firing for an extended period. This sustained activity acts as a memory of the negative event. In this context, excessive integration between the OFC and the DMN could signify an over-representation of negative emotional and motivational information within the self-referential DMN. This could lead to a persistent, negative, and self-focused bias that contributes significantly to the clinical experience of negative symptoms. From this perspective, the observed therapeutic effect in our study, the improvement of negative symptoms, would be a result of the intervention helping to uncouple this pathological non-reward activity from the DMN, thereby alleviating the persistent negative bias and its associated symptoms."

Reference:

Rolls ET. A non-reward attractor theory of depression. Neurosci Biobehav Rev. 2016;68:47-58. doi:10.1016/j.neubiorev.2016.05.007

- Methods: "The clinical staff and patients were blind to the assignment, except for one clinical technician who provided the rTMS or sham treatment according to the randomization numbers." - this sentence appears in two sections, and should be eliminated from "Participants"

Response: We appreciate the reviewer's careful reading and valuable feedback. We have removed the duplicate sentence from the "Participants" section.

- Methods: "with the coil vertically positioned over the AF8 site": more clarity is needed: does this mean that the handle was downwards, so that the coil current was directed upwards, at right angles to the line of the head circumference, as in Feffer et al. or Fettes et al among the references? The exact orientation and direction of the coil and current should be indicated.

Response: We thank the reviewer for this valuable question regarding the details about coil which is essential for replication. In our study, we indeed followed the protocols described by Fettes et al (2017; PMID: 28017321). We have now added these crucial details to the Methods section to ensure clarity and reproducibility.

Methods section [Page 19, Line 17-24]

"Similar to the previous study [19], the intervention was administered using a MagPro ×100 stimulator equipped with a 120 º-angled figured 9 coil (Cool D-B80) and a coil cooler unit (MagVenture). In the active rTMS group, the stimulation was targeted to the right orbital frontal cortex with the coil vertex positioned over the AF8 site, which was defined according to the international 10–10 EEG system. The coil was oriented with the handle vertical and upwards, perpendicular to the axial plane of the head, and current flow directed superiorly, for optimal stimulation of the horizontal shelf of the OFC."

Reference:

Fettes P, Peters S, Giacobbe P, Blumberger DM, Downar J. Neural correlates of successful orbitofrontal 1 Hz rTMS following unsuccessful dorsolateral and dorsomedial prefrontal rTMS in major depression: A case report. Brain Stimul. 2017;10(1):165-167. doi:10.1016/j.brs.2016.11.008

- Methods: were there any scalp electrodes placed near the coil to mimic the scalp sensations in the sham group? These electrodes are controversial and not necessarily required, but it should be made explicit as to whether they were present or not. Was a MagVenture sham coil used (if so, what model number) or was it a custom-built apparatus?

Response: Thank you for raising these important points regarding our sham procedure. We agree that providing these details is critical for replication and transparency.

As the reviewer notes, we did place scalp electrodes near the coil in both the active and sham groups. The primary purpose of these electrodes was to record TMS-EEG signals to explore changes in cortical-evoked activity, as described in our previous work (Jiao et al., 2024, PMID: 38852897). These electrodes were not used to mimic scalp sensations.

Regarding the sham coil: We used same coil (Cool D-B80) in the sham condition. The same protocol was conducted for the sham group except that the coil was flipped 180º so it produced the same "click" sound but was unable to produce a magnetic field.

We have now added these details in the Methods section as below:

Results section [Page 19, Line 29-33]

"The same protocol and same coil was conducted for the sham group except that the coil was flipped 180º so it produced the same "click" sound but was unable to produce a magnetic field. Besides, scalp electrodes were placed in both groups to record TMS–EEG activity. These electrodes were not used to mimic scalp sensations."

Reference:

Jiao X, Hu Q, Tang Y, et al. Abnormal Global Cortical Responses in Drug-Naïve Patients With Schizophrenia Following Orbitofrontal Cortex Stimulation: A Concurrent Transcranial Magnetic Stimulation-Electroencephalography Study. Biol Psychiatry. 2024;96(5):342-351. doi:10.1016/j.biopsych.2024.05.024

Reviewer #2:

This manuscript was generally well-written. It's a well-designed RCT to explore the efficacy and neural mechanisms of 1Hz rTMS over the right OFC in first-episode, drug-naïve people with schizophrenia. It combined with rs-fMRI and proposed a multi-level causal pathway analysis. It's a timely contribution to the current topics of precision medicine in psychiatry. I have several comments/suggestions for further possible improvement of the manuscript.

1. After demonstrating the DMN differences, the authors distinguished between the anterior and posterior parts of the DMN for analysis. Given the location of rTMS stimulation, why is it not consider to compare between left and right sides? It may be clinically reasonable.

Response: We appreciate the reviewers' suggestions. We initially partitioned the DMN into anterior and posterior sections for our analysis. This decision was based on established functional distinctions within the network, which is often considered to comprise two primary subsystems: an anterior subsystem centered on the medial prefrontal cortex (mPFC) and a posterior subsystem centered on the posterior cingulate cortex (PCC) (e.g., Yeo et al., 2011; PMID: 21653723).

Following this analysis, we also considered the left-right hemispheric division as proposed. The ANOVA results showed that while the group differences for both the left ($F=7.544, p=0.007$) and right ($F=6.636, p=0.012$) sides were statistically significant, this categorical approach had limited explanatory power. This prompted us to re-examine the issue from a

more continuous perspective: whether the strength of the neural effect induced by rTMS correlates with the physical distance of each ROI from the stimulation site.

As shown in Fig. 2 E-G, our results confirmed this distance-based relationship. Therefore, we suggest that although the physiological effects of TMS generally follow a distance-decay pattern at a macro level, they do not show significant hemispheric lateralization. This reveals a degree of independence between functional and structural architectures at the local scale (e.g., Honey et al, 2009; PMID: 19188601). This phenomenon likely reflects that the influence of TMS is transmitted via complex, polysynaptic functional pathways rather than being strictly limited by direct anatomical adjacency. Therefore, in the revised version, we present the anterior and posterior DMN results in the main text and include the left-right hemispheric analyses in the supplementary figures for clarity and completeness.

The revised Results section now reads:

Results section [Page 7, Line 24-27]

"We also conducted a left–right hemispheric division but found no evidence of right-sided dominance. Significant group differences were observed in both hemispheres (left: $F(1,82) = 7.544$, $p = 0.007$, *partial* $\eta^2 = 0.08$; right: $F(1,82) = 6.636$, $p = 0.012$, *partial* $\eta^2 = 0.07$; see Supplementary Fig. S1)."

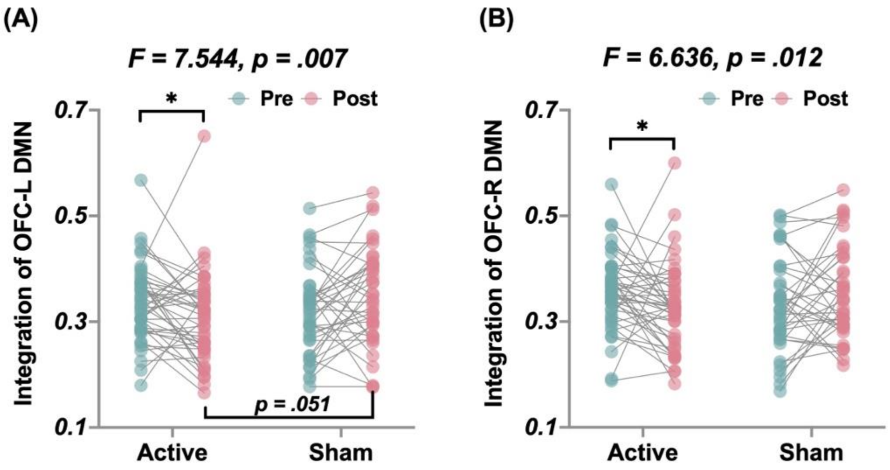

Fig. S1 Integration coefficients changes between OFC and the left DMN (A), and right DMN (B). Gray lines connect data points from the same subject, illustrating individual changes between pre-intervention and post-intervention. Dots represent integration coefficients in the pre-intervention (green) and in the post-intervention (red). ANOVA analysis showed significant group differences in both hemispheres: left, $F(1,82) = 7.544$, $p = 0.007$, *partial* $\eta^2 = 0.08$, and right, $F(1,82) = 6.636$, $p = 0.012$, *partial* $\eta^2 = 0.07$, with reduced OFC–DMN integration in the active group but not in the sham group after rTMS

treatment.

Reference:

Honey CJ, Sporns O, Cammoun L, et al. Predicting human resting-state functional connectivity from structural connectivity. Proc Natl Acad Sci U S A. 2009;106(6):2035-2040. doi:10.1073/pnas.0811168106

Yeo BT, Krienen FM, Sepulcre J, et al. The organization of the human cerebral cortex estimated by intrinsic functional connectivity. J Neurophysiol. 2011;106(3):1125-1165. doi:10.1152/jn.00338.2011

2.In the previous rTMS studies, high frequency parameters (eg. 5Hz rTMS targeting for left DLPFC) were often employed. It's especially reported for the improvement of emotional symptoms. In the present trial (a novel combination of parameter and targeting area), the two arms were 1Hz rTMS and sham control over the right OFC, is there any emotional symptom related variable recorded?

Response: We appreciate the reviewer's insightful question regarding the assessment of emotional symptoms and the rationale for our specific rTMS protocol. In our study, our primary outcomes were based on the PANSS and MCCB, which are widely used in schizophrenia trials. Besides, we also include assessment of emotional symptoms as reflected in the PANSS Depressed Subscale, the Hamilton Depression Scale (HAMD), and the Hamilton Anxiety Scale (HAMA). As you rightly point out, our results (in Table1 and clinical symptoms result section) show a significant group by session interaction for depressed symptoms, which post-hoc analysis revealed a significant improvement in the PANSS depressed subscale ($t(82)=-2.64$, $p=0.047$), whereas we did not find significant changes in the total scores of the HAMD or HAMA.

This discrepancy between the PANSS and Hamilton scales is an important finding, and it could reflect differences in their psychometric properties. The PANSS depressed subscale is a clinician-rated measure specifically designed to assess depression in the context of schizophrenia, accounting for the overlap between depressive and negative symptoms, making it more sensitive to changes in this population. In contrast, the HAMD and HAMA are broader depression and anxiety scales, respectively, which may not adequately capture the specific emotional changes that occur in current population.

Therefore, our results suggest that although our intervention may not have been powerful enough to elicit a significant change on a global measure of depression (HAMD), it was indeed effective in targeting the specific depressive symptoms that are intertwined with the psychopathology of schizophrenia. We believe this finding is a crucial nuance that supports

the therapeutic potential of our rTMS protocol for a broader range of symptoms, including those related to emotional regulation.

We have added this detailed explanation in Discussion and to provide a more nuanced interpretation of our clinical findings in implication section as below:

Results section [Page 17, Line 21-25]

"Besides, the significant improvement observed in the PANSS depressed subscale, despite the lack of change in the broader Hamilton scales, suggests that specific emotional symptoms that are associated with schizophrenia psychopathology may improve alongside improvements in other symptoms. This highlights the potential of our intervention to address the complex emotional dysregulation often present in this populations."

3.Please provide the trial registration number;

Response: We thank the reviewer for their request to provide the trial registration number. To ensure full transparency and compliance with reporting guidelines, we can confirm that the trial registration number has been included in the manuscript. The trial registration number is [ChiCTR2000041106].

For the reviewer's convenience, we have highlighted its location in the manuscript. It can be found in the:

Method section under Participants [Page 19, Line 1-3]

"The study was approved by the Ethics Committee of Shanghai Mental Health Center and The First Psychiatric Hospital of Harbin and registered with the Chinese Clinical Trial Register Center (Registration number: ChiCTR2000041106). All participants provided informed consent".

4.It needs detailed evidence based description or critical comment, eg. In the second paragraph of background part, TMS has played 'an invaluable role';

Response: We thank the reviewer for this helpful suggestion. To provide stronger evidence for our statement, we have now added supporting references in the Background section:

Introduction section [Page 3, Lines 11–12]

"Transcranial magnetic stimulation (TMS), a noninvasive brain stimulation technique, has played an invaluable role in modulating brain activity and clinical symptoms [10–14]."

Reference:

10. Aleman, A., Enriquez-Geppert, S., Knegtering, H. & Dlabac-de Lange, J. J. Moderate effects of noninvasive brain stimulation of the frontal cortex for improving negative symptoms in schizophrenia: Meta-analysis of controlled trials. Neuroscience and Biobehavioral Reviews vol. 89 111–118 Preprint at https://doi.org/10.1016/j.neubiorev.2018.02.009 (2018).

11. Beynel, L., Powers, J. P. & Appelbaum, L. G. Effects of repetitive transcranial magnetic stimulation on resting-state connectivity: A systematic review. Neuroimage 211, (2020).

12. Lorentzen, R., Nguyen, T. D., McGirr, A., Hieronymus, F. & Østergaard, S. D. The efficacy of transcranial magnetic stimulation (TMS) for negative symptoms in schizophrenia: a systematic review and meta-analysis. Schizophrenia 8, (2022).

13. Marzouk, T., Winkelbeiner, S., Azizi, H., Malhotra, A. K. & Homan, P. Transcranial Magnetic Stimulation for Positive Symptoms in Schizophrenia: A Systematic Review. Neuropsychobiology vol. 79 384–396 Preprint at https://doi.org/10.1159/000502148 (2020).

14. Wobrock, T. et al. Left prefrontal high-frequency repetitive transcranial magnetic stimulation for the treatment of schizophrenia with predominant negative symptoms: A sham-controlled, randomized multicenter trial. Biol Psychiatry 77, 979–988 (2015).

5.In the viewpoint of public mental health, I'd like to recommend use people with schizophrenia instead of 'patient with schizophrenia' in the manuscript.

Response: We thank the reviewer for this thoughtful and important suggestion. We fully agree that using "people with schizophrenia" is more appropriate from a public mental health perspective.

We have revised the manuscript throughout to reflect this change, now using "people with schizophrenia" instead of "patients with schizophrenia." Additionally, we have used the term "subjects" where appropriate to refer to patients in the study.

Reviewer #3:

Zeng et al investigate rTMS-induced effects on OFC connectivity and its relationship to

symptomatic and cognitive improvements in patients with schizophrenia. The authors conduct a range of interesting analyses and offer a comprehensive model of the mechanistic pathway for the effectiveness of OFC stimulation in the disorder.

A few points of clarification on the methods are warranted; aside from this I have mostly minor comments.

Introduction

- The introduction is generally well written, but would benefit from some additional proof-reading to improve on some somewhat vague phrases in several places (e.g. “noninvasive transcranial brain technique”; “causal structure of brain and behavioral symptoms”)

Response: We appreciate the reviewer’s feedback on the Introduction. We have carefully proofread the entire section and revised several phrases to improve clarity and precision.

Specifically, we have replaced “noninvasive transcranial brain technique” with the more precise term “noninvasive brain stimulation technique”. Additionally, we have refined the phrase “causal structure of brain and behavioral symptoms” to “modulating brain activity and clinical symptoms” to better introduce transcranial magnetic stimulation (TMS). Now the revised sentence reads as:

Introduction section [Page 3, Line 11-12]

“Transcranial magnetic stimulation (TMS), a noninvasive brain stimulation technique, has played an invaluable role in modulating brain activity and clinical symptoms [10–14].”

Reference:

10. Aleman, A., Enriquez-Geppert, S., Knegtering, H. & Dlabac-de Lange, J. J. Moderate effects of noninvasive brain stimulation of the frontal cortex for improving negative symptoms in schizophrenia: Meta-analysis of controlled trials. Neuroscience and Biobehavioral Reviews vol. 89 111–118 Preprint at https://doi.org/10.1016/j.neubiorev.2018.02.009 (2018).

11. Beynel, L., Powers, J. P. & Appelbaum, L. G. Effects of repetitive transcranial magnetic stimulation on resting-state connectivity: A systematic review. Neuroimage 211, (2020).

12. Lorentzen, R., Nguyen, T. D., McGirr, A., Hieronymus, F. & Østergaard, S. D. The efficacy of transcranial magnetic stimulation (TMS) for negative symptoms in schizophrenia: a systematic review and meta-analysis. Schizophrenia 8, (2022).

13. Marzouk, T., Winkelbeiner, S., Azizi, H., Malhotra, A. K. & Homan, P. Transcranial Magnetic Stimulation for Positive Symptoms in Schizophrenia: A Systematic Review. Neuropsychobiology vol. 79 384–396 Preprint at https://doi.org/10.1159/000502148

(2020).

14. Wobrock, T. et al. Left prefrontal high-frequency repetitive transcranial magnetic stimulation for the treatment of schizophrenia with predominant negative symptoms: A sham-controlled, randomized multicenter trial. Biol Psychiatry 77, 979–988 (2015).

- It would be interesting to understand a little more about what is known of the clinical efficacy and network dynamics of OFC stimulation outside of the current study. Could the authors briefly expand on any findings in other datasets investigating the effects of OFC rTMS, if available?

Response: We appreciate the reviewer's suggestion for additional context on the clinical efficacy and network dynamics of OFC stimulation, which we agree is essential for highlighting the novelty of our study. We have now added a brief overview of relevant findings in the Introduction section.

Introduction section [Page 3, Line 33-42 to Page 4, Line 1-4]

"A series of studies have demonstrated encouraging results for OFC-rTMS. Inhibitory OFC-rTMS has shown promise in the treatment of obsessive-compulsive disorder (OCD) and substance use disorders (SUD), with studies reporting associated reductions in activation of disorder-relevant brain regions [20,21]. Notably, Fettes et al. (2017) first reported a case of major depression resistant to conventional rTMS over the dorsolateral prefrontal cortex (DLPFC) and the dorsomedial prefrontal cortex (DMPFC) but showed marked improvement in mood, anxiety and anhedonia following 1 Hz right OFC-rTMS, accompanied by a reduction in functional connectivity between the OFC and the nucleus accumbens [18]. Subsequent work has further substantiated the safety, tolerability, and clinical outcomes of this protocol in a review [19]. This raises the question whether OFC-induced physiological effects can be used to reliably improve symptoms and cognitive function in SCZ. Our recent TMS/EEG study demonstrated a reduced EEG response to right OFC stimulation in SCZ[25]. However, the mechanistic effects on network modularity remain to be investigated."

Reference:

18. Fettes, P., Peters, S., Giacobbe, P., Blumberger, D. M. & Downar, J. Neural correlates of successful orbitofrontal 1 Hz rTMS following unsuccessful dorsolateral and dorsomedial prefrontal rTMS in major depression: A case report. Brain Stimulation vol. 10 165–167 Preprint at https://doi.org/10.1016/j.brs.2016.11.008 (2017).

19. Feffer, K. et al. 1 Hz rTMS of the right orbitofrontal cortex for major depression: Safety,

tolerability and clinical outcomes. European Neuropsychopharmacology 28, 109–117 (2018).

20. Hanlon, C. A. et al. What goes up, can come down: Novel brain stimulation paradigms may attenuate craving and craving-related neural circuitry in substance dependent individuals. Brain Res 1628, 199–209 (2015).

21. Nauczyciel, C. et al. Repetitive transcranial magnetic stimulation over the orbitofrontal cortex for obsessive-compulsive disorder: a double-blind, crossover study. Transl Psychiatry 4, e436–e436 (2014).

25. Jiao, X. et al. Abnormal Global Cortical Responses in Drug-Naïve Patients With Schizophrenia Following Orbitofrontal Cortex Stimulation: A Concurrent Transcranial Magnetic Stimulation–Electroencephalography Study. Biol Psychiatry 96, 342–351 (2024).

Methods and results

- Could the authors expand on why negative correlations in the dFC matrices were set to 0?

Response: Thank you for this important question. Our analytical approach is situated within the graph theoretical framework commonly used in network neuroscience (Duch W, 2020, PMID: 32415206; Garcia JO, 2018, PMID: 30559531; Khambhati AN, 2018, PMID: 28645844). Within this framework, brain regions are quantified as nodes, and the edges between them are considered to reflect their coordinated activity, which is quantified using measures of time-series synchrony. The modularity analysis we employ aims to understand how these nodes form densely interconnected groups, or modules, and how these modules are organized into a potential hierarchy across the whole-brain network.

In this context, positive dFC has a more direct and well-established neurophysiological interpretation, representing the strength of synergy between ROIs. In contrast, negative dFC is often interpreted as reflecting antagonistic or competitive interactions, with less straightforward implications in many graph-theoretic analyses, such as community detection. The manner in which these antagonistic relationships are processed in defining a cohesive functional module remains to be elucidated. Therefore, to ensure a more tractable and neurophysiologically grounded interpretation of network modules, we excluded negative connections from our primary analysis.

Reference:

Finc K, Bonna K, He X, et al. Dynamic reconfiguration of functional brain networks during working memory training. Nat Commun. 2020;11(1):2435. Published 2020 May 15.

doi:10.1038/s41467-020-15631-z

Garcia JO, Ashourvan A, Muldoon SF, Vettel JM, Bassett DS. Applications of community detection techniques to brain graphs: Algorithmic considerations and implications for neural function. Proc IEEE Inst Electr Electron Eng. 2018;106(5):846-867. doi:10.1109/JPROC.2017.2786710

Khambhati AN, Sizemore AE, Betzel RF, Bassett DS. Modeling and interpreting mesoscale network dynamics. Neuroimage. 2018;180(Pt B):337-349. doi:10.1016/j.neuroimage.2017.06.029

- Could the authors expand on how corrections for multiple comparisons were applied? The methods state that "P values of group comparison was corrected by Bonferroni" – is this referring to the fact that group x session interactions are investigated for connectivity between OFC and 8 different networks? Further,10 NNMF components are tested for the interaction of interest – are corrections applied at this level, too? Similarly, the "Data analysis" section later simply states that "Multiple comparison used Bonferroni correction", which is insufficient information as it is unclear what (how many models) this refers to.

Response: We thank the reviewer for pointing out details that we overlooked. In the current study, Bonferroni correction was used in the ANOVA analysis for both the OFC-based integration coefficients and NMF components. Specifically, for OFC-based integration coefficients, $p_{Bonferroni} = 0.05/8$, whereas for NMF components, $p_{Bonferroni} = 0.05/10$. We have added this in the Data Analysis section and reported the adjusted p-values in the Results section.

1）Results section [Page 6, Line 33-35]

"A mixed-design ANOVA analysis showed that "the interaction between group and session on integration between right OFC and DMN was significant (Fig. 2A), $F(1,82) = 7.872$, $p_{Bonferroni} = 0.048$, *partial* η^2=0.09".

2）Results section [Page 9, Line 10-12]

"Notably, the 10th component dominated by the ventromedial prefrontal cortex (vmPFC; Fig. 3B) exhibited a significant group × session interaction ($F(1, 82) = 8.09$, $p_{Bonferroni} = 0.05$, *partial* η^2=0.09)."

3）Methods section [Page 24, Line 28-30]

"Bonferroni correction was used to control false positives for the ANOVA results for both integration coefficients (p = 0.05/8) and NMF components (p = 0.05/10)."

- It is not entirely clear what motivated the specific focus on the attention network. Presumably this is based on findings related to attention/vigilance (AV) scores. The authors appear to be using "cognitive alertness" as a conceptual synonym for AV – this will need to be explicitly mentioned in order to make the link to the attention network analyses. In addition, the statement "Cognitive alertness is a common manifestation of positive symptoms and tends to be more connected to the attention network" needs refining – it is unclear in how far cognitive alertness is a "manifestation" of positive symptoms, and please provide a reference to its (intuitive) link to the attention network.

Response: We appreciate the reviewer for pointing out the imprecise term "cognitive alertness" and the link between "cognitive alertness" and "positive symptoms".

As the reviewer speculated, the exploratory analysis of the attention network in this study was based on the findings related to attention/vigilance (AV) scores from MATRICS Consensus Cognitive Battery (MCCB). Regarding the expression "cognitive alertness", we must admit that this is an imprecise description of the term "attention/vigilance", and we apologize for this. We have changed the term "cognitive alertness" to "attentional alertness" throughout the revised manuscript and can further link to the attention network analysis.

The attentional alerting system is often used to describe a state of heightened arousal initiated when an individual anticipates an upcoming stimulus and is closely related to the attention network (e.g., Langner R, 2013, PMID: 23163491; Markett S, 2022, PMID: 34882908). In the context of schizophrenia, AV actually reflects cognitive deficits rather than direct positive symptoms. We believe that attentional alertness and positive symptoms may be indirectly related, as positive symptoms are characterized by abnormal salience processing and hallucinations (René S Kahn, 2015, PMID: 27189524).

In general, we agree with the reviewers and have made the following changes to the manuscript: First, we changed the term "cognitive alertness" to "attentional alertness" throughout the text and the title. Second, for "Cognitive alertness is a common manifestation of positive symptoms and tends to be more connected to the attention network", we revised the statement and provided relevant references as below:

Manuscript title [Page 1, Line 1-2]

"OFC-induced network modularity improves positive symptoms and attentional alertness in schizophrenia: a combined rTMS-fMRI Study"

Results section [Page 12, Line 24-25]

"Attentional alertness may reflect abnormal salience processing in positive symptoms[34],

and tend to be more connected to the attention network than the DMN[35,36]."

Reference:

34. Kahn, R. S. et al. Schizophrenia. Nat Rev Dis Primers 1, 15067 (2015).

35. Langner, R. & Eickhoff, S. B. Sustaining attention to simple tasks: A meta-analytic review of the neural mechanisms of vigilant attention. Psychol Bull 139, 870–900 (2013).

36. Markett, S., Nothdurfter, D., Focsa, A., Reuter, M. & Jawinski, P. Attention networks and the intrinsic network structure of the human brain. Hum Brain Mapp 43, 1431–1448 (2022).

- The statistical language is at time imprecise, e.g. "interaction of integration between the right OFC and DMN between group and session was significant" – do the authors mean "the interaction between group and session on integration between right OFC and DMN was significant"?

Response: We appreciate the reviewer's careful attention to the precision of our statistical language. We agree that our original phrasing, "interaction of integration between the right OFC and DMN between group and session was significant" was imprecise.

In the revised manuscript, we have corrected this and similar expressions to adopt a more accurate and standard formulation:

Results section [Page 6, Line 33-34]

"The interaction between group and session on integration between the right OFC and DMN was significant."

Minor comments:

- "with matched the age and sex" occurs several times in the manuscript, please correct

Response: This has now been corrected. We deleted "with matched the age and sex" in the Method section and now only appear in the "participants" in Result section.

- The input to NMF is described as the "difference of integration coefficients" – please be explicit that this refers to the difference between pre and post treatment

Response: We thank the reviewer for pointing out this ambiguity. We apologize for the unclear description. In our study, for each subject's pre- and post-intervention data, the

integration coefficients between the OFC and 50 nodes within the DMN were used as inputs to NMF. We have revised the sentence to read as follows:

Results section [Page 9, Line 6-8]

"Here, for all subjects, the integration coefficients between the OFC and 50 nodes within the DMN at two time points (pre-session and post-session) were used as the inputs for NMF."

- Fig 4 legend typo: "mediaction"

Response: We have corrected this typo, and the Fig. 4 legend now reads:

Figure legend [Page 12, Line 2–7]

"Fig. 4 | Cognitive function mediates, and positive symptoms moderate the effects of OFC-DMN modularity." …(B) Mediation analysis results."

Reviewer #4 :

This study aimed to provide a more comprehensive understanding of how brain stimulation functions under repetitive transcranial magnetic stimulation (rTMS) treatment. The authors seek to transform rTMS from a "black-box" intervention into a mappable, observable therapeutic process, supported by direct visual evidence of network-level effects.

The exceptional novelty lies in the mechanistic transparency achieved through dynamic community detection methods. Researchers and clinicians observed how brain networks reorganize during treatment, watching therapeutic effects propagate both spatially and temporally from the stimulation site through connected brain systems. The dynamic integration coefficient visualizations provide insights into therapeutic network reconfiguration, showing how orbitofrontal cortex (OFC) stimulation reduces pathological integration with the default mode network (DMN) over time. This level of mechanistic visualization has never been demonstrated in previous rTMS-OFC studies and represents a paradigm shift toward mechanism-guided therapy.

The visual biomarkers showed here might enable treatment monitoring, allowing clinicians to track patient responses by observing network integration patterns during treatment. The

distance-dependent effect provide a spatial map of treatment influence, potentially guiding optimal coil placement and stimulation parameters. The integration of genetic expression data with treatment-responsive brain regions offers a novel framework for personalized medicine, where baseline patterns might predict treatment response.

The choice of orbitofrontal cortex as a stimulation target represents a significant innovation, departing from conventional dorsolateral prefrontal and temporoparietal approaches. The authors demonstrate that OFC stimulation produces spatially constrained effects that cascade through specific network pathways, ultimately improving both positive symptoms and cognitive function. The comprehensive analytical framework spanning genes, networks, cognition, and symptoms shows how modern neuroscience methods can be integrated to understand complex treatment mechanisms at multiple biological levels. Their demonstration that reduced OFC-DMN integration corresponds with clinical improvements offers a concrete therapeutic target for future treatment development. The temporal dynamics suggest that network-based biomarkers could optimize stimulation protocols and predict individual outcomes.

The fundamental contribution of making rTMS mechanisms visible and quantifiable represents a transformative advance establishing new standards for understanding neuromodulation effects. However, important technical limitations warrant consideration in future clinical applications.

Major Concerns

1) The central premise of this study, reduced OFC-DMN integration represents beneficial "modularity", requires more substantial theoretical and empirical justification. Throughout the manuscript, the authors consistently interpret decreased integration as therapeutically advantageous, assuming a direct causal pathway from reduced integration to improved modularity to better clinical outcomes. However, this interpretation rests on several unexamined assumptions that weaken the overall argument. The paper would benefit from addressing why reduced integration should be considered inherently beneficial rather than potentially reflecting network disruption or dysfunction. Without healthy control comparisons to establish baseline integration levels, it remains unclear whether the observed OFC-DMN integration in patients is indeed pathologically excessive or within normal ranges. The authors should consider that optimal brain function may require balanced rather than minimal integration, and that the therapeutic effects could result from normalization rather than simple reduction of connectivity.

Response: We appreciate the reviewer’s constructive feedback. We agree that the manuscript would be strengthened by a more nuanced discussion of why reduced OFC-DMN integration is considered therapeutically beneficial in the context of SCZ.

Our premise is based on a substantial body of literature indicating that hyperactivation and hyperconnectivity of the DMN are hallmark features of schizophrenia pathophysiology (Whitfield-Gabrieli S et al., 2009, PMID: 19164577; Whitfield-Gabrieli S & Ford JM, 2012, PMID: 22224834). This aberrant DMN activity is linked to core symptoms, including self-referential processing deficits that may contribute to paranoid ideation and hallucinations, as well as cognitive impairments in attention and working memory. As we mentioned in the Discussion, excessive integration between the OFC and DMN may blur the boundary between internal thoughts and the external environment, leading to an exaggerated focus on self-referential processing. Therefore, we believe it is more plausible to interpret the reduced integration observed after rTMS treatment as a therapeutic modulation of a pre-existing state of hyperconnectivity.

We agree with the reviewer that optimal brain function requires balanced, not minimal, integration and that the therapeutic effect is likely a result of normalization rather than a simple reduction. We have revised the manuscript to reflect this more precise interpretation.

We also acknowledge the reviewer's point regarding the lack of a healthy control group to establish baseline integration levels. This is a limitation of our study design, which was a randomized controlled trial focused on treatment effects within a patient population. We have now explicitly stated this limitation in the manuscript's "Limitations" section.

We have made the following revisions to the manuscript to address these points:

1) Discussion section [Page 15, Line 21-39]

"A major finding of this study is that 4 weeks of low-frequency rTMS treatment significantly reduced the dynamic integration between the OFC and DMN in people with schizophrenia. These findings are encouraging as extensive literature has documented the DMN to be implicated in spontaneous cognitive activity and self-referential processes [6,37,38]. Its hyperactivation and hyperconnectivity may be considered hallmark features of schizophrenia, contributing to the observed reduced modularity of network[39]. A study in people at ultra-high risk for psychosis examined resting state connectivity of the salience network (SN) and DMN in association with facial emotion recognition [40]. They report increased connectivity between the SN and the medial prefrontal cortex, an area of the DMN, in participants at ultra-high risk for psychosis. Our results show that the putatively normalizing effect of rTMS was particularly pronounced in a component dominated by the vmPFC, a core hub of the DMN. Anatomically, the OFC relays multi-sensory information to downstream structures via the vmPFC, playing a crucial role in regulating emotion and motivation[22,41]. Therefore, the excessive integration between the OFC and DMN observed in psychosis-risk states may blur the boundary between internal thoughts and external reality. This disruption leads to an exaggerated focus on self-referential thoughts, where

neutral events are imbued with personal significance—a mechanism believed to underpin core symptoms like paranoid ideation and hallucinations [6,42]. By reducing this aberrant integration, our rTMS protocol may help restore a more balanced and modular network architecture."

2) Limitations Section [Page 17, Line 30-34]

"This study has several limitations. First, our study lacks a healthy control group. Without establishing baseline network integration levels in a non-clinical population, we cannot definitively conclude that the observed reduction in OFC-DMN integration represents a normalization of pathologically excessive connectivity. Future studies including a healthy control group would be crucial to confirm this interpretation."

References:

Whitfield-Gabrieli S, Thermenos HW, Milanovic S, et al. Hyperactivity and hyperconnectivity of the default network in schizophrenia and in first-degree relatives of persons with schizophrenia. Proc Natl Acad Sci U S A. 2009;106(4):1279-1284. doi:10.1073/pnas.0809141106

Whitfield-Gabrieli S, Ford JM. Default mode network activity and connectivity in psychopathology. Annu Rev Clin Psychol. 2012;8:49-76. doi:10.1146/annurev-clinpsy-032511-143049

2) The study presents a significant logical inconsistency between its mediation and moderation analyses that undermines the coherence of the proposed mechanism. The mediation analysis suggests that cognitive improvements mediate the relationship between brain connectivity changes and symptom improvement, positioning cognition as an intermediate step in a linear causal chain. However, the moderation analysis indicates that positive symptoms moderate the relationship between brain integration changes and attention improvements, suggesting an interactive rather than mediating role for symptoms. These findings represent fundamentally different causal models that cannot both be correct simultaneously. The authors acknowledge this discrepancy in lines 411-417 but fail to provide a satisfactory resolution or explanation. This inconsistency raises questions about the stability and interpretability of the observed relationships and suggests the need for more careful theoretical modeling of how brain changes, cognitive improvements, and symptom reductions interact following rTMS intervention.

Response: We appreciate the reviewer's comments. The results of mediation and moderation analyses do point to different causal models, a complex phenomenon. After re-examining these findings, we believe that the two models may not be in stark contradiction but rather

may reflect different dimensions of the complex systemic properties of rTMS intervention. We acknowledge that no single statistical model can fully capture the dynamic and potentially interactive relationships between the brain, cognition, and symptoms.

Therefore, we attempt to propose a speculative explanatory framework that aims to reconcile these two analytical findings. We speculate that this discrepancy may stem from different levels of analysis: First, the mediation analysis captures a “macro” or “global” level of change. At this level, the overall cognitive improvement induced by rTMS may be a primary, yet relatively simplified, pathway for overall symptom improvement. Second, the moderation analysis may reveal a conditional relationship at the “micro” or “specific” level. This may indicate that, within the context of these macro trends, the efficiency of a specific neural pathway (OFC-DMN) in translating into a specific cognitive function (attention) is dynamically influenced by the underlying state of positive symptoms.

Based on this line of thought, perhaps cognition is indeed a key pathway for symptom improvement, but this pathway is not static, and the “degree of patency” of certain specific links within it is modulated by symptom status. Of course, These exploratory statistical models alone cannot establish a firm causal relationship between cognition and symptoms, and this is just a theoretical speculation based on existing data. Our primary conclusions remain based on the RCT design itself, namely, the causal effect of the intervention on each outcome variable.

To address this issue, we have revised the limitations section to clarify the relevant speculation.

Limitations section [Page 17, Line 42 to Page 18, Line 1-13]

“Finally, discrepancies existed between the mediation and moderation analyses, which warrants careful interpretation. While these findings might seem to represent contradictory causal models, we speculate that they may not be mutually exclusive: they may capture phenomena at different analytical levels. The mediation model, using global scores, may reflect a macro-level pathway where broad cognitive improvements contribute to overall symptom reduction. The moderation model, using specific variables, could be revealing micro-level dynamics, where the translation of specific neural gains into improved attention is conditional upon the patient's positive symptom state. Under this hypothetical framework, it is possible that while cognitive enhancement is a primary pathway to recovery (mediation), the efficiency of this pathway for certain domains is dynamically regulated by concurrent symptoms (moderation). These exploratory analyses underscore the complex, likely bidirectional, interplay between cognitive and symptomatic changes following rTMS. We propose this not as a definitive conclusion, but as a testable hypothesis for future research.”

3) The genetic association analysis, while intriguing, suffers from post-hoc reasoning that limits its mechanistic interpretability. The authors correlate gene expression maps with treatment-induced changes in brain integration patterns and conclude that the observed effects are "supported by underlying excitatory neurotransmission and expression of schizophrenia-related genes." However, this approach demonstrates correlation rather than causation and doesn't establish that genetic factors predict treatment response or drive the therapeutic mechanisms. The temporal logic is problematic: genetic expression patterns are used to explain treatment-induced changes, but no evidence is provided that these genetic factors influenced treatment response prospectively. The analysis would be more appropriately framed as exploratory validation of the biological plausibility of the findings rather than mechanistic evidence for the treatment effects.

Response: We appreciate and acknowledge the reviewer's concern. The current presentation of the genetic association analysis could falsely imply a causal or mechanistic role, which is beyond the scope of our data.

This analysis is correlational in nature, revealing spatial overlap between treatment-responsive brain network patterns and specific gene expression profiles, rather than a causal relationship. We agree with the reviewer that this analysis was exploratory and that there is no evidence that these genetic factors can prospectively predict individual responses to intervention.

Therefore, we have revised the relevant sections of the manuscript, repositioning this analysis as an exploratory one aimed at verifying the biological plausibility of our neuroimaging findings. The emphasis of our current discussion is that, by linking macroscopic brain network changes to microscopic molecular biological context, we provide cross-scale convergent evidence that the observed treatment effects are rooted in biological processes relevant to the pathophysiology of SCZ.

1) Abstract section [Page 2, Line 11-13]

"The spatial topography of this pattern was correlated with the expression of schizophrenia-related genes and markers of excitatory neurotransmission, supporting its biological relevance."

2) Results section one [Page 9, Line 21-25]

"To investigate the biological plausibility of this rTMS-sensitive integration pattern, we performed an exploratory analysis correlating its spatial topography with transcriptional maps from the Allen Human Brain Atlas. The goal was to determine if the brain regions most modulated by the treatment were enriched for genes known to be relevant to

schizophrenia pathophysiology."

3) Results section two [Page 9, Line 38-42]

"Together, these findings suggest that the NMF10 component, a key integration pattern modulated by rTMS, is spatially aligned with a molecular landscape implicated in schizophrenia. This correlation provides convergent, cross-scale evidence for the biological relevance of our neuroimaging findings, though it does not imply a causal relationship."

4) Discussion section [Page 16, Line 11-19]

"Furthermore, our exploratory analysis of gene expression patterns adds biological plausibility to this network-level finding. The spatial map of the vmPFC-dominated integration pattern was significantly correlated with the expression of genes linked to schizophrenia risk. Specifically, this map was enriched for genes related to excitatory neurons, synaptic function, and signal transduction [44,45]. This spatial association does not imply that these genes mechanistically drive the treatment response, but rather provides important, cross-scale validation. The large-scale network dynamics modulated by our rTMS protocol are rooted in the specific molecular and cellular systems long implicated in the pathophysiology of schizophrenia."

4) The Granger causality analysis presents additional methodological concerns that may compromise the validity of the mechanistic interpretations. The authors apply Granger causality to integration coefficients derived from dynamic network measures rather than to raw time series data, which may not reflect true temporal causality relationships. Furthermore, the interpretation that reduced causal influence from OFC-DMN to DMN-Attention networks is beneficial could alternatively indicate breakdown of important regulatory mechanisms. The limited temporal resolution (156 time windows from 4-minute scans) may be insufficient for robust causality inference, particularly for the complex, derived measures used in this analysis. The authors should provide stronger justification for this analytical approach and consider alternative interpretations of reduced causality that don't assume therapeutic benefit.

Response: We appreciate the reviewer's comments. Applying Granger causality analysis to derived indicators is a nonstandard approach that requires careful justification and interpretation. We need to clarify the theoretical assumptions and exploratory nature of this analysis.

First, we want to clarify that the purpose of this analysis was not to explore causal relationships between individual brain regions, in which case using raw BOLD time series would undoubtedly be standard practice. In fact, our analysis was a targeted, confirmatory one designed to test a specific network-level hypothesis: does the DMN act as a "relay station" in

the information flow from the OFC to the AN? In other words, do changes in upstream (OFC-DMN) connectivity dynamics induced by TMS intervention drive or causally influence changes in downstream (DMN-AN) connectivity dynamics? To address this high-order question regarding the interplay between these “edges”, we chose to use the time series of integration coefficients, representing the dynamic strength of these two “edges”, as input to our Granger causality analysis. We believe this is an appropriate approach to directly test our “relay” or “cascade effect” hypothesis, even though it is certainly an exploratory one.

Second, we agree that the interpretation of the reduced causal effect requires caution. Our initial interpretation was based on two considerations: 1) this reduction in effect is intuitively consistent with the physiological effects of 1Hz rTMS (an inhibitory stimulus); and 2) it is consistent with our core hypothesis that reducing excessive DMN interference with downstream AN is beneficial. Meanwhile, we acknowledge another possibility: this reduced causal effect could also reflect the disruption of an important regulatory mechanism. We performed correlation analyses between Granger causality and intervention effects but found no significant results. Therefore, our data cannot distinguish between these two possibilities, and interpreting this finding as a clear “treatment benefit” would be biased. In the revised manuscript, we have adjusted the wording to interpret this as a preliminary finding consistent with the clinical improvement we observed. In addition, we address the alternative explanations pointed out by the reviewers and emphasize that further studies are needed to clarify their precise biological significance.

Finally, the temporal resolution of 156 time windows is indeed a significant limitation for robust causal inference. This limits the strength and generalizability of our conclusions. Therefore, we framed this Granger causality analysis as an exploratory, hypothesis-driven preliminary test rather than a definitive conclusion. We have added this explanation in the limitations section of the manuscript.

1) Results section [Page 12, Line 24-26 to Page 13, Line 1-26]

“Attentional alertness may reflect abnormal salience processing in positive symptoms[34], and tend to be more connected to the attention network than the DMN[35,36]. One potential explanation is that the DMN may act as a relay, transmitting the intervention effect to the downstream attention network. To explore this specific, network-level hypothesis, we employed Granger causality analysis in an exploratory capacity. Critically, our aim was not to infer causality between brain regions, but rather to test whether dynamic fluctuations in the upstream network coupling (OFC-DMN integration) causally influenced the fluctuations in downstream network coupling (DMN-AN integration). Accordingly, we used the time series of the integration coefficients for these two network pairs as inputs for the Granger causality analysis.

Mixed-design ANOVA analysis showed ……In addition, we observed that the reduced causal influence may be affected by the extreme values of the active group in the baseline. We therefore excluded the two subjects in the active group whose pretest causal influence exceeded 0.1 and repeated the analysis with the remaining subjects. The results still showed a significant interaction ($F = 4.352$, $p = 0.040$, *partial* η^2=0.05), and the reduction of Granger causality in the active group was close to significant ($t = 1.950$, $p = 0.058$, *Cohen's d* = 0.29, see supplementary Fig.S4). After accounting for potential regression to the mean, our analysis provides preliminary evidence suggesting that the rTMS-induced modularity between the OFC and DMN may attenuate the DMN's causal influence on the downstream AN. Although this attenuation is consistent with the inhibitory effects of 1-Hz rTMS and our overall hypothesis, we should interpret this finding with caution: this could also reflect the disruption of a potentially important regulatory mechanism, a possibility that our data cannot exclude."

2) Discussion section [Page 15, Line 9-16]

"Encouraged by this, we further focused on specific intervention pathways and found that alterations in brain integration selectively improved attentional performance, with this improvement being moderated by positive symptoms. Exploratory Granger causality analysis provided preliminary evidence for a potential mechanism: the causal influence from the OFC-DMN pathway to the downstream DMN-AN pathway was reduced in the active group. While requiring cautious interpretation, these finding hint that rTMS may work by decoupling the DMN from its downstream antagonistic networks."

3) Limitations section [Page 17, Line 40-42]

"Fourth, the limited temporal resolution afforded by 156 time windows may be insufficient for robust causality inference. These Granger causalities should be viewed as preliminary, exploratory findings."

5) Several aspects of the statistical analysis raise concerns about the reliability and generalizability of the findings. The study examines multiple brain networks (8 networks), numerous cognitive domains, and various symptom subscales, creating substantial multiple comparison issues that are only partially addressed through Bonferroni correction for network-level analyses. The complex moderation analyses are conducted on a relatively small active treatment group (n=45), which may be underpowered to detect reliable interaction effects, particularly given the number of variables examined. The authors report p-values extensively but provide limited information about effect sizes, making it difficult to assess the clinical significance of the observed changes. Additionally, the 4-week intervention period with immediate post-treatment assessment provides no information about the stability or

durability of the observed effects, limiting conclusions about therapeutic utility.

Response: We agree that this is an important concern, regarding the rigor of the statistical analysis and study design. We would like to provide more detailed clarification of our analytical strategy and the limitations of our study.

First, we understand the reviewer's concern about multiple comparisons. We would like to clarify that for all primary hypothesis tests in this study, namely, mixed-design ANOVAs involving eight brain networks and the ten NMF components, we rigorously used Bonferroni correction for multiple comparisons and report corrected p-values in the results. Some subsequent exploratory analyses were designed to further explore positive findings from the primary analyses. For example, our moderator analysis was driven by a clear a priori hypothesis: in our correlational network analysis, we observed significant associations between three specific variables: brain (OFC-DMN integration), cognition (attention/alertness), and symptoms (positive symptoms). Thus, the moderator analysis specifically examined interactions between these three pre-screened variables, rather than conducting untargeted exploration across a wide range of variables. This, mitigated the problem of multiple comparisons.

Second, we agree that caution is warranted when conducting moderation analyses with a sample size of 45. To ensure the robustness of our results, we employed bootstrap analysis to test the significance of the moderation effect. This is a widely recommended, more robust statistical inference method when sample size is limited or data do not conform to a perfect normal distribution. Nevertheless, we acknowledge that these findings should be considered preliminary and warrant further verification of their robustness in larger studies.

Third, reporting only p-values in the manuscript is indeed insufficient to fully assess the significance of the results. In response to your suggestion, we have supplemented the manuscript with corresponding effect size indicators for the main statistical analyses so that readers can better assess the magnitude and clinical significance of the changes we observed.

Finally, the lack of long-term follow-up data is a limitation of this study. We cannot draw conclusions about the durability of treatment effects beyond the 4-week intervention period. We would like to add that at the end of the intervention, we used the Global Assessment of Functioning (GAF) and the Clinical Global Impressions (CGI) to assess patients' overall functional status. As shown in Table 1, both measures showed significant group × time interaction effects, indicating that the intervention resulted in clinically meaningful, global improvements in functioning at the end of treatment. Although this is not a substitute for long-term follow-up, it provides support for the immediate effects of the intervention.

We thank the reviewer again for the detailed comment, which greatly helped us improve the quality of the manuscript. We have added these details in revised manuscript:

Results section, add effect size [Page 6, Line 33-41 to Page 7, Line 1-4]

"A mixed-design ANOVA analysis showed that the interaction between group and session on integration between right OFC and DMN was significant (Fig. 2A), $F(1,82) = 7.872$, $p_{Bonferroni} = 0.048$, ***partial*** $\boldsymbol{\eta^2=0.09}$. Post-hoc analysis found that, after 4 weeks of rTMS treatment (Fig. 2B), integration between the right OFC and DMN was significantly reduced in the active group ($t(82) = 2.15$, $p = 0.035$, ***Cohen's d*** **=0.24**), whereas no such effect was observed in the sham group ($t(82) = -1.83$, $p = 0.070$, ***Cohen's d*** **= 0.20**). To further explore the specifity of this effect, we subdivided the DMN into anterior DMN and posterior DMN (Fig. 2C & 2D). We found that anterior DMN exhibited a stronger treatment response ($F(1,82) = 9.165$, $p = 0.003$, ***partial*** $\boldsymbol{\eta^2=0.10}$). Integration between the OFC and anterior DMN was significantly decreased in the active group after rTMS treatment ($t(82) = 2.57$, $p = 0.012$, ***Cohen's d*** **= 0.28**), whereas no such effect was observed in the sham group ($t(82) = -1.75$, $p = 0.084$, ***Cohen's d*** **= 0.19**). We also conducted a left–right hemispheric division but found no evidence of right-sided dominance. Significant group differences were observed in both hemispheres (left: $F(1,82) = 7.544$, $p = 0.007$, ***partial*** $\boldsymbol{\eta^2 = 0.08}$; right: $F(1,82) = 6.636$, $p = 0.012$, ***partial*** $\boldsymbol{\eta^2 = 0.07}$; see Supplementary Fig. S1).

Results section, add effect size [Page 9, Line 10-15]

Notably, the 10th component dominated by the ventromedial prefrontal cortex (vmPFC; Fig. 3B) exhibited a significant group × session interaction ($F(1, 82) = 8.09$, $p_{Bonferroni} = 0.05$, **partial** $\boldsymbol{\eta^2=0.09}$)." Post-hoc analysis revealed a significant reduction in integration within the active group following rTMS treatment ($t(82) = 2.32$, $p = 0.023$, **Cohen's d = 0.26**), whereas no significant change was observed in the sham group ($t(82) = -1.73$, $p = 0.087$, **Cohen's d = 0.19**)."

Limitation [Page 17, Line 38-40]

"Furthermore, the lack of long-term follow-up means the stability of these improvements remains an important question that needs to be answered in future studies."

6) The paper lacks evidence for the specificity of the observed effects to OFC stimulation, which weakens claims about the unique therapeutic potential of this target. Without comparison to other stimulation sites, it remains unclear whether the observed network changes represent OFC-specific mechanisms or general effects of prefrontal stimulation. The distance-dependent effects described in the results are presented as supporting evidence for OFC-specific influence, but the underlying mechanism for why proximity should determine integration changes is not adequately explained. The authors should acknowledge this limitation or provide stronger theoretical and empirical support for OFC-specific mechanisms.

Response: We appreciate the reviewer's comments. We agree that demonstrating the specificity of effects is crucial for understanding the OFC as a therapeutic target. The analytical pipeline in this study was designed to specifically examine the effects of the right OFC as a target for intervention. Therefore, our analyses, including the calculation of network integration, were conducted using the OFC as a "seed." A key finding supporting the specificity of our conclusions is the group-level distance-dependent effect reported in the Results section: the closer the physical distance between the DMN region and the OFC target, the greater the reduction in its integration. This suggests that the effects of the intervention spread outward from the OFC stimulation target.

To further strengthen empirical support for this finding, we conducted a new supplemental analysis. We calculated the correlation between the change in integration (post-pre/post+pre) and the distance to the OFC target at the individual level and compared the correlation coefficients between the two groups. The results showed that this distance-dependent effect was significantly stronger in the active group than in the sham group, reaching borderline significance (t-test: active > sham, $t = 1.944$, $p = 0.055$). This individual-level finding further validated the distance-dependent effect observed at the group level and provided additional support for the conclusion that the stimulation effect originated from the OFC target. We have incorporated this new result into the manuscript.

Furthermore, we acknowledge that the lack of a control group for stimulation of an alternative prefrontal location in this study limited our ability to assess the specific associations between OFC and improvements in cognition and symptoms. This is an important consideration, which we have addressed in the Discussion section of the manuscript.

We revised the manuscript as follows:

1) Results section [Page 7, Line 22-27]

"We also compared the correlations of distance-dependent effects between the two groups at the individual level. A two-sample t-test showed marginal significance (active > sham, $t = 1.944$, $p = 0.055$, see supplementary Fig.S2). Although it did not reach significance, combined with the group-level correlation findings, this to some extent indicates that OFC-induced physiological effects indeed reduce integration with the DMN in SCZ, and such intervention effect decreases with increasing brain distance."

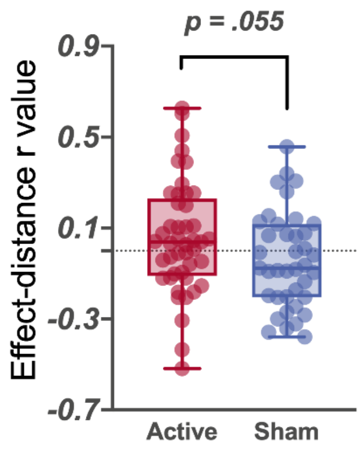

Fig. S2 Distance-dependent effects between the active and sham group at the individual level. Dots represent correlation r values between intervention effect and Euclidean distance in the active group (red) and sham group (blue). A two-sample t-test showed marginal significance (t=1.94, p=0.055).

2) Discussion section [Page 17, Line 17-21]

"The observed distance-dependent effects provide evidence that our stimulation protocol engaged the intended OFC-centered circuitry. However, it is important to acknowledge that, in the absence of comparison with other stimulation sites, the extent to which the observed clinical effects are unique to OFC stimulation remains an important open question."

7) The theoretical framework connecting reduced OFC-DMN integration to improved attention and reduced positive symptoms requires more rigorous development. The paper assumes that DMN hyperactivation interferes with attention networks and that reducing this interference is beneficial, but this simplified model may not capture the complexity of real brain network interactions. Networks are not purely competitive systems, and reducing communication between them could impair rather than improve function. The authors should consider alternative models where optimal function requires balanced rather than minimal inter-network communication and address how their findings fit within broader theories of network dysfunction in schizophrenia.

Response: We appreciate this theoretical suggestion, which prompted us to conduct a more comprehensive literature review to interpret our findings. We agree that, for interventions in psychiatric disorders, optimal brain function should stem from a balance, not minimization, of inter-network communication. A common finding in schizophrenia is that the DMN often exhibits hyperactivity and hyperconnectivity (Looijestijn J et al., 2015, PMID: 26432501; Whitfield-Gabrieli S, Ford JM, PMID: 22224834). This hyperactive state of the DMN is

thought to be associated with excessive self-referential processing and an inability to divert attentional resources from internal thoughts, leading to impairments in cognitive functions such as attention and working memory. Therefore, our interpretation is grounded in this "imbalance" theory. We speculate that the reduced OFC-DMN integration observed in the study is not simply a "functional inhibition", but rather a "normalization" of the pathological "imbalance".

To more precisely elucidate this process, we integrated our findings into a recently proposed three-dimensional model of neural activity and phenomenal-behavioral patterns (Martino M, Magioncalda P, 2024, PMID: 38844531). This model is based on the classical phenomenological concepts of perception (related to environmental stimuli) and imagery/concept (independent of environmental stimuli), providing a new perspective for understanding psychotic symptoms. It posits that a core neural basis for psychotic symptoms lies in the combined effects of sensory system dysfunction and hyperactivity in DMN regions. In this imbalance, an overactive DMN sends excessive top-down feedback signals to the weakened sensory cortex, resulting in unusually vivid internal experiences unrelated to external environmental input and persistent beliefs that are difficult to correct with external evidence.

Within this exploratory framework, our findings can be theorized. The OFC may be attenuating this pathological, top-down "associative drive" by regulating the function of the core nodes of the DMN. When this excessive internal information flow originating from the DMN is normalized, the overall balance of the brain network is reset. This allows the AN, responsible for processing external tasks, to be less disrupted by spontaneous DMN activity, allowing more cognitive resources to be allocated to the external world. This provides a more complete and biologically plausible explanation for the observed improvements in attention and reductions in positive symptoms.

We now have revised the Discussion section of the manuscript to provide a more in-depth explanation of our findings based on this improved theoretical framework.

Disscussion section [Page 16, Line 41-42 to Page 17, Line 1-12]

"Recently, a working model of neural activity and phenomenal experience proposed that DMN hyperactivity (the "associative unit" in the model) may drive aberrant top-down signals to sensory cortices, leading to a dissociation of phenomenal experience from the external environment, which manifests as positive symptoms like hallucinations and delusions[51]. From this perspective, the reduced OFC-DMN integration observed following our rTMS intervention may not represent a simple suppression of function, but rather a therapeutic normalization of this pathological DMN hyperactivity. By modulating an anterior hub of the DMN, the intervention may dampen this excessive top-down

“associative drive”. This rebalancing of large-scale network dynamics would, in turn, restore a healthier state of segregation between the internally-focused DMN and the externally-focused AN. The subsequent reduction of DMN’s influence over the AN could free up crucial cognitive resources, allowing for improved attentional control and a diminished intrusion of the aberrant internal experiences that constitute positive symptoms. Exploratory Granger causality finding of OFC-DMN-AN pathway, provides preliminary support for this proposed mechanism.”

References:

Looijestijn J, Blom JD, Aleman A, Hoek HW, Goekoop R. An integrated network model of psychotic symptoms. Neurosci Biobehav Rev. 2015;59:238-250. doi:10.1016/j.neubiorev.2015.09.016

Martino M, Magioncalda P. A working model of neural activity and phenomenal experience in psychosis. Mol Psychiatry. 2024;29(12):3814-3825. doi:10.1038/s41380-024-02607-4

Whitfield-Gabrieli S, Ford JM. Default mode network activity and connectivity in psychopathology. Annu Rev Clin Psychol. 2012;8:49-76. doi:10.1146/annurev-clinpsy-032511-143049

8) The authors should provide more conservative interpretations of their correlation-based findings, particularly regarding genetic associations and network causality. The study would benefit from acknowledging that observed associations establish relationships rather than mechanisms and that the proposed causal chains require additional validation. Future studies could strengthen these claims by including healthy control groups for baseline comparisons, longer follow-up periods to assess stability, and direct comparisons with other stimulation targets to establish specificity.

Response: We thank the reviewer’s suggestions. We agree that the findings based on the correlation analysis should be interpreted more rigorously and conservatively. We have carefully revised the manuscript to ensure that our interpretations are consistent with the exploratory nature of the data:

First, the revised manuscript now explicitly states that genetic association analyses are intended to provide “biological plausibility” for neuroimaging findings and that they reveal spatial correlations rather than causal mechanisms. Similarly, we discuss the methodological limitations of the Granger causality analysis and offer alternative interpretations of the results.

Second, in the Limitations section of the manuscript, we acknowledge the lack of a healthy control group, which limits our ability to draw conclusive conclusions about the

“normalization” effect. We also point out that the absence of alternative stimulation targets limits our ability to infer OFC target specificity. In response to your suggestion, we have added a discussion regarding the lack of long-term follow-up.

We believe that with these revisions, the manuscript presents a more balanced and prudent presentation of the findings and will also provide clearer ideas for subsequent studies in this field.

Now, the Limitations section reads as follow:

Limitations section [Page 17, Line 30-42 to Page 18, Line 1-13]

“This study has several limitations. First, our study lacks a healthy control group. Without establishing baseline network integration levels in a non-clinical population, we cannot definitively conclude that the observed reduction in OFC-DMN integration represents a normalization of pathologically excessive connectivity. Future studies including a healthy control group would be crucial to confirm this interpretation. Second, although we observed an improvement in overall cognitive scores, no specific cognitive domain showed distinct enhancements, limiting the scope of conclusions about specific cognitive benefits. Third, our inferences about OFC treatment specificity are also limited, given the absence of other stimulation targets for comparison. Furthermore, the lack of long-term follow-up means the stability of these improvements remains an important question that needs to be answered in future studies. Fourth, the limited temporal resolution afforded by 156 time windows may be insufficient for robust causality inference. These Granger causalities should be viewed as preliminary, exploratory findings. Finally, discrepancies existed between the mediation and moderation analyses, which warrants careful interpretation. While these findings might seem to represent contradictory causal models, we speculate that they may not be mutually exclusive: they may capture phenomena at different analytical levels. The mediation model, using global scores, may reflect a macro-level pathway where broad cognitive improvements contribute to overall symptom reduction. The moderation model, using specific variables, could be revealing a micro-level dynamics, where the translation of specific neural gains into improved attention is conditional upon the patient's positive symptom state. Under this hypothetical framework, it is possible that while cognitive enhancement is a primary pathway to recovery (mediation), the efficiency of this pathway for certain domains is dynamically regulated by concurrent symptoms (moderation). These exploratory analyses underscore the complex, likely bidirectional, interplay between cognitive and symptomatic changes following rTMS. We propose this not as a definitive conclusion, but as a testable hypothesis for future research.”

9) The figure legends throughout the manuscript lack sufficient detail for independent interpretation and fail to meet the standards expected for a high-impact journal. Many legends assume readers are familiar with the specific methodologies and abbreviations used, making the figures difficult to understand without repeatedly referencing the methods section. The legends should be comprehensive enough that readers can understand the main findings from the figures alone, following the principle that figures should be self-contained units of scientific communication. The figure legends consistently fail to provide fundamental information necessary for independent interpretation. Technical terms and abbreviations are used without definition, leaving readers to guess at meanings or search through the methods section for clarification. Color schemes, symbols, and scaling information are routinely omitted, making it impossible to interpret the visual data being presented. Sample sizes are rarely specified for individual analyses, and when statistical significance indicators are used, their meanings and thresholds are not explained. Units of measurement are frequently absent from quantitative displays, and perhaps most importantly, the legends provide no biological or clinical interpretation of what significant findings actually mean in the context of the research question. This lack of comprehensive detail forces readers to constantly cross-reference between figures, methods, and results sections, significantly reducing the accessibility and impact of the visual presentations. The legends should serve as complete, self-contained explanations that allow readers to understand both the technical aspects of the analysis and the scientific significance of the findings without requiring extensive background reading.

For example: Figure 2: The current legend simply states "group × session interaction of integration in 8 networks" without explaining what "integration" means in this context, how it was calculated, or what the color coding represents. The legend should specify that integration refers to "the average probability that the OFC is in the same community as nodes from each network across time windows" and explain that higher values indicate greater cross-network communication. The violin plots in panels B-D lack explanation of what the individual points represent (are these individual subjects or time windows?), and the color scheme is not defined. The correlation plots in panels E-G need clearer explanation of what "Euclidean distance" refers to (maybe physical distance in mm between brain regions? This even did not mention in Methods) and why this relationship is theoretically important. I cannot find the relevant descriptions in Results or Discussions.

Response: We thank the reviewer for this constructive comment regarding the figure legends. Following the suggestion, we have comprehensively revised all figure captions in the revised manuscript to ensure they are self-contained and allow independent interpretation. The improvements are as follows:

First, we have clearly defined key methodological terms (e.g., “non-negative matrix factorization”, “Granger causality”) and abbreviations (e.g., NMF, GC) at the relevant

locations of each figure legend.

Next, we explained the contents and visual elements of figures. For each figure, we explicitly describe the chart types used (e.g., violin plots, scatter plots), and the meaning of the colors, symbols, and scaling. Group (Active/Sham) and time point (Pre/Post) information are clearly annotated in the legends.

Third, we have supplemented concise methodological explanations where necessary. For example, in the caption to Figure 2, we defined the meaning of "Euclidean distance" (i.e., the physical distance between the OFC target and the coordinates of the ROIs in various DMN brain regions, measured in millimeters) and explained the theoretical importance of testing this relationship (i.e., examining whether the physiological effects of rTMS are spatially specific). We have also supplemented this definition of "Euclidean distance" in the Methods section for consistency.

Finally, we uniformly indicate the p-value threshold for statistical significance marks (such as asterisks *) at the end of the figure legends and provide a brief biological or clinical interpretation of the results to help readers understand their scientific significance.

Together, these changes ensure that the figures can serve as stand-alone units of scientific communication The main changes are list as follows:

1) Results section [Page 7, Line 11-13]

"The distance was defined as the linear physical distance (in millimeters) between each DMN ROI and the center coordinates of the right OFC stimulation target in the MNI space."

2) Figure legends

Fig. 1 | Study design and data analysis pipeline [Page 5, Line 8-16 to Page 6, Line 1-5]:

(A) Flowchart of the randomized controlled trial (RCT) design. Individuals with schizophrenia (SCZ) were randomized to receive either 4 weeks of active 1Hz repetitive transcranial magnetic stimulation (rTMS) targeting the right orbitofrontal cortex (OFC) or a sham stimulation. Clinical symptoms were assessed at baseline, 2 weeks, and 4 weeks. Cognitive function and resting-state fMRI were assessed at baseline and 4 weeks. (B) Schematic of the neuroimaging data processing and analysis pipeline. Resting-state fMRI time series were extracted from Power 264 atlas and OFC target. Dynamic functional connectivity matrices were constructed using a sliding time-window approach. A multi-layer community detection algorithm was then applied to these matrices to calculate the integration of OFC with different networks, which quantifies the probability that two ROIs from different networks are assigned to the same community over time; higher values

indicate greater cross-network communication. These brain-level metrics were then integrated with symptom, cognitive, and transcriptomic data for statistical analysis.

Fig. 2 | Low-frequency rTMS reduces dynamic integration between OFC and default mode network [Page 8, Line 2-23]:

(A) Statistical results for the group × session interaction for integration between the OFC and eight canonical brain networks. Integration refers to "the average probability that the OFC is in the same community as nodes from each network across time windows". Higher values indicate greater cross-network communication. Eight distinct colors denote eight brain networks. A significant interaction was observed only for the Default Mode Network (DMN), surviving Bonferroni correction. (B-D) Plots show the changes in integration coefficients between OFC and the whole DMN (B), the anterior DMN (aDMN; C), and the posterior DMN (pDMN; D). Blue violin represents values of pre-intervention and red violin represents values of post-intervention. Dashed lines represent the medium and quartiles. Gray lines connect data points from the same subject, illustrating individual changes between pre-intervention and post-intervention. (E-G) Scatter plots showing the relationship between OFC-DMN integration and Euclidean distance. Euclidean distance is the physical distance in MNI space (mm) between each DMN ROI and the OFC stimulation target. This analysis tests the hypothesis that rTMS effects are spatially constrained. (E) At baseline, a negative correlation exists, indicating that more distant ROIs are less integrated with OFC. (F) After treatment, this relationship is abolished in the active group. (G) The change in integration (ΔOFC-DMN) is positively correlated with distance in the active group, indicating that the greatest reduction in integration occurred in ROIs closest to the stimulation target. Red dots represent values of active group, blue dots represent values of sham group, and shaded areas represent 95% confidence intervals (95%CI). In all panels, * denotes $p < 0.05$, uncorrected.

Fig. 3 | A vmPFC-dominated integration pattern is modulated by rTMS and is spatially correlated with schizophrenia-related gene expression [Page 10, Line 2-18].

(A) Schematic of non-negative matrix factorization (NMF), a data-driven method used to decompose the subject-by-ROI integration matrix into a set of spatial components (W) and their corresponding subject-level weights (H). (B) NMF identified 10 components. Component 10, spatially dominated by the ventromedial prefrontal cortex (vmPFC), showed a significant group × session interaction. The bar chart shows the weights for all 10 components, and the line plot illustrates the significant post-treatment reduction in the weight of component 10 in the active group compared to the sham group. Word clouds

represent cognitive terms associated with the Component 10. (C-F) Exploratory analysis showing the spatial correlation between the Component 10 map and various gene expression profiles from publicly available databases. This analysis assesses the biological plausibility of the component. (C) The component's topography is correlated with genes associated with schizophrenia-like psychosis. (D) It is enriched for excitatory neuronal cell-type markers (Ex4, Ex5). (E) Gene expression patterns are associated with developmental stages of the OFC and related regions. (F) Genes are enriched in biological processes related to synaptic function and signal transduction. In all panels, * denotes $p < 0.05$, uncorrected.

Fig. 4 | Cognitive function mediates and positive symptoms moderate the effects of OFC-DMN modularity [Page 12, Line 2-22].

(A) Inter-subject correlation (ISC) analysis. ISC matrices show the similarity between every pair of subjects (active group) based on their treatment-induced changes in brain integration, cognitive performance, and clinical symptoms. Higher values (warmer colors) indicate greater similarity. (B) Mediation analysis results. The model shows that the effect of brain integration changes on symptom improvement is fully mediated by changes in cognitive function. (C) Correlation network of specific sub-domains. The graph illustrates significant correlations ($p < 0.05$, uncorrected) between treatment-induced changes in specific brain (red), cognitive (green), and symptom (blue) measures. Line thickness corresponds to the strength of the correlation. (D) Moderation analysis model. The model tests whether the effect of OFC-DMN integration changes on attention/vigilance (AV) is conditional upon the level of positive symptom change (F3 at 2 weeks). (E) Simple slope plot of the moderation effect. The relationship between reduced OFC-DMN integration (more modularity) and improved AV performance is significant only when there is a reduction in positive symptoms (low ΔF3(2w), solid line). Statistical significance is indicated as **$p < 0.01$, uncorrected. PANSS: positive and negative syndrome scale. F1: negative factor. F2: disorganized factor. F3: positive factor. F4: excited factor. F5: depressed factor. CGI: clinical global impression. GAF: global assessment function. TMT: trail making test. BACSS: brief assessment of cognition schizophrenia: symbol coding. AV: attention/vigilance. WM: working memory. RPS: reasoning and problem-solving.

Fig. 5 | rTMS attenuates the causal influence from the pathway OFC-DMN-attention network [Page 14, Line 2-13].

(A) Granger causality (GC) was used to test the hypothesis that dynamic changes in OFC-DMN coupling causally influence downstream DMN-Attention Network (AN) coupling.

Among the seven downstream networks, significant interactions were only observed in the DMN-AN pathway. (B) Group-average time series of integration coefficients for the OFC-DMN (red) and DMN-AN (blue) pathways, before and after the intervention for both active (top) and sham (bottom) groups. Shaded areas represent the standard error of the mean. (C) Line plot showing the strength of GC from the OFC-DMN to the DMN-AN pathway. The active group shows a significant post-treatment reduction in causal influence, an effect not observed in the sham group. Individual gray lines connect data points from the same subject. In all panels, * denotes $p < 0.05$, uncorrected.

For ease of reference, all modifications made in the text are **highlighted in orange**.

Reviewer #2:

The authors replied all the questions and comments, and revised it accordingly. There were two suggestions for minor revision:

1.In the abstract, the conclusion should revised more conservative interpretations accordingly. “the critical role” - (“These findings reveal the critical role for the dynamic modularity of the OFC as an “intermediate phenotype” mediating the pathway from genetic variation to behavioral manifestations, ”)

We agree with the reviewer that the term “critical” was too strong. To address this concern, we revised the abstract by replacing “the critical role” with more conservative wording. Specifically, “critical role” was changed to “important role”.

Page 2, Line 19:

These findings reveal the important role for the dynamic modularity of the OFC as an “intermediate phenotype” mediating the pathway from genetic variation to behavioral manifestations.

2.In the background part: In the second paragraph of background part, TMS has played ‘an invaluable role’, from the related evidence, TMS for schizophrenia doesn’t reach ‘an invaluable role’, it’s better to use other proper word/expression.

We agree with the reviewer that the term “invaluable” overstated the current evidence base of TMS in schizophrenia. Accordingly, we revised this expression and replaced “an invaluable role” with “a promising role,” which more accurately reflects the current level of evidence in the literature.

Page 3, Line 12:

Transcranial magnetic stimulation (TMS), a noninvasive brain stimulation technique, has played a promising role in modulating brain activity and clinical symptoms.
